# Comparative Transcriptome Analyses between a Spontaneous Late-Ripening Sweet Orange Mutant and Its Wild Type Suggest the Functions of ABA, Sucrose and JA during Citrus Fruit Ripening

**DOI:** 10.1371/journal.pone.0116056

**Published:** 2014-12-31

**Authors:** Ya-Jian Zhang, Xing-Jian Wang, Ju-Xun Wu, Shan-Yan Chen, Hong Chen, Li-Jun Chai, Hua-Lin Yi

**Affiliations:** 1 Key Laboratory of Horticultural Plant Biology, Ministry of Education, Huazhong Agricultural University, Wuhan, 430070, China; 2 National Key Laboratory of Crop Genetic Improvement, Huazhong Agricultural University, Wuhan, 430070, China; 3 Kunming Academy of Agricultural Sciences, Kunming, 650000, China; 4 Engineering Technology College, Huazhong Agricultural University, Wuhan, 430070, China; USDA-ARS-SRRC, United States of America

## Abstract

A spontaneous late-ripening mutant of ‘Jincheng’ (*C. sinensis* L. Osbeck) sweet orange exhibited a delay of fruit pigmentation and harvesting. In this work, we studied the processes of orange fruit ripening through the comparative analysis between the Jincheng mutant and its wild type. This study revealed that the fruit quality began to differ on 166^th^ days after anthesis. At this stage, fruits were subjected to transcriptome analysis by RNA sequencing. 13,412 differentially expressed unigenes (DEGs) were found. Of these unigenes, 75.8% were down-regulated in the wild type, suggesting that the transcription level of wild type was lower than that of the mutant during this stage. These DEGs were mainly clustered into five pathways: metabolic pathways, plant-pathogen interaction, spliceosome, biosynthesis of plant hormones and biosynthesis of phenylpropanoids. Therefore, the expression profiles of the genes that are involved in abscisic acid, sucrose, and jasmonic acid metabolism and signal transduction pathways were analyzed during the six fruit ripening stages. The results revealed the regulation mechanism of sweet orange fruit ripening metabolism in the following four aspects: First, the more mature orange fruits were, the lower the transcription levels were. Second, the expression level of *PME* boosted with the maturity of the citrus fruit. Therefore, the expression level of *PME* might represent the degree of the orange fruit ripeness. Third, the interaction of PP2C, PYR/PYL, and SnRK2 was peculiar to the orange fruit ripening process. Fourth, abscisic acid, sucrose, and jasmonic acid all took part in orange fruit ripening process and might interact with each other. These findings provide an insight into the intricate process of sweet orange fruit ripening.

## Introduction

Bud mutants have always been applied as the genetic materials for the studies of molecular mechanism in the plant field. *Citrus* species have various bud mutants in fruit color [1], seed number [2], fruit sterility [3], and flowering time [4]. These mutants contribute to both breeding and the understanding of biological processes such as pigment metabolism, wax biosynthesis, seedlessness mechanism, and self-incompatibility mechanism. There are also many mutants in *Citrus* fruit ripening including some mutants in fruit color. The ‘Tardivo’ mandarin, as a late ripening mutant of the ‘Comune’ clementine (*Citrus clementina* Hort. Ex Tanaka), was analyzed physiologically and genetically, demonstrating the involvement of ethylene in the regulation of at least some aspects of peel maturation [5], [6]. Mingliutianju (*Citrus reticulat*a *Blanco cv*. Mingliutianju), as a late-ripening mutant of Chuntianju, was analyzed at the transcriptome level, revealing 18 different biological processes including flavonols' metabolism. And these 18 processes may be related to its mutation [7]. The ‘Fengwan’ orange (*Citrus sinensis* L. Osbeck), as a late ripening mutant of the ‘Fengjie 72-1’ orange, was analyzed at the transcriptome and proteome levels during three fruit ripening stages, indicating the importance of sucrose and abscisic acid to fruit ripening [8].

RNA sequencing (RNA-seq) is an effective and popular method for transcriptional analysis and has been used for the mining of differentially expressed genes, alternative splicing, and SNP detection [9]. With the reduced cost of RNA-seq, it has been frequently used to study dynamic biological processes in fungi [10], plants [11] and animals [12]. In addition, RNA-seq is highly accurate in quantifying expression levels, which can be determined by quantitative PCR, and RNA-seq exhibits high levels of reproducibility for both technical and biological replicates [13], [14]. A large number of data obtained by RNA-seq present a macroscopic yet detailed view of transcriptome of the samples. However, data mining is an onerous and time-consuming task.

Fruit ripening is a complex process that involves sugar accumulation, acid degradation, carotenoid accumulation, and fruit softening, etc [15]. Besides, fruit ripening is also a flexible programme [16]. According to the Web of Knowledge database 2013, most researches on the fruit ripening process focused on climacteric fruits, researches on which were almost five times as many as those on non-climacteric fruits. Many substances such as transcription factors, plant hormones and microRNA have been reported to involve in the regulation of fruit ripening process. Recently, a study of tomato fruit ripening identified a fruit specifically expressed enzyme β-D-N-acetylhexosaminidase (β-Hex), and proved that RIN could directly or indirectly regulate the transcription of β-Hex through SIASR during fruit ripening [17]. A research on Chinese pear ripening by using RNA-seq, reported that ABA, auxin, GA and BR could also regulate fruit ripening by interacting with ethylene, and that the members of MADS, NAC, WRKY and HSF family could regulate fruit ripening at a transcriptome level [18]. FUL, a MADS family member was found regulating tomato fruit ripening by fine-tuning ethylene biosynthesis and ripening-related genes expression [19]. Most of the studies of *Citrus* fruit ripening were focused on pigmentation [20], [21], [22] and hormones [23], [24], by studying natural mutants or materials under physical or chemical treatments. With the help of next generation sequencing, a microRNA, Csi-miR164, and its function in fruit ripening stage was identified and was validated to target a NAC transcription factor [25]. It is well known that abscisic acid (ABA) is a most important hormone functioning in the ripening process of non-climacteric fruit [26], [27]. Sucrose also participates in the grape [28] and strawberry [29], [30] ripening processes. Some researches reported that MeJA promoted the ripening of strawberry by affecting anthocyanin accumulation, cell wall modification and the biosynthesis of ethylene and JAs [31]. While other researches reported that MeJA could slow down or inhibite the ripening of strawberry fruit [32]. Overall, there has not been sufficient information about the specific ripening mechanism of citrus fruit so far.

In this work, we analyzed the external and internal quality of the fruit of ‘Jincheng’ sweet orange in six fruit ripening stages. Fruits at 166 days after anthesis (DAA) were subjected to RNA-seq analysis to detect the differences between the wild type and its late ripening mutant at the overall transcriptome level. The significantly enriched groups of the differentially expressed unigenes (DEGs) were subjected to secondary classification. The expression profiles of the majority of the genes that are involved in ABA, sucrose and JA metabolism and the signal transduction pathway were analyzed for the first time throughout the citrus fruit ripening process. The work provides new information on citrus fruit ripening.

## Materials and Methods

### Plant material and sample collection

A wild type ‘Jincheng’ sweet orange (WT) and its spontaneous late-ripening mutant (MT), which were cultivated in the same orchard in Yunpan Village, Xingshan County, Yichang City, Hubei Province, China, were used in this research. We get the permission of citrus fruits' collection from the authority of the Bureau of Specialty in Xingshan, and the field studies did not involve endangered or protected species. Fruit samples of wild genotype were collected from three trees, and the samples of mutant genotype were from other three trees. These three mutant sampling trees were propagated from the same original mutant and grafted on the same kind of rootstock. Twelve representative fruits were sampled from each tree at each time point. Altogether, there were 36 sampling fruits (12*3) representing each genotype at each time point. These samples were collected at six time points from September to December: respectively at 139, 166, 182, 199, 215 and 232 DAA. Six fresh fruits out of 36 sample fruits were used for color measurement. The pulps of the rest 30 fruits were separated from the peel, and then were cut into cubes and mixed. These treated samples were immediately frozen in liquid nitrogen, and kept at −80°C for RNA extraction and the determination analyses of the composition and concentration of soluble sugar and organic acid.

### Color index determination

The color variation of the *Citrus* peel was measured with a MINOLTA CR-400 chromameter (Japan) by the CIELAB color system. Twenty-four points on the surface of six fruits (four points for one fruit) were measured for each sample per time point. The presented values are the color index values (CI = 1000 a/Lb; L, 0 to 100, black to white; a, ± yellow/blue; b, ± red/green), in which green and orange colors are represented by negative and positive values, respectively [33]. Student's t test (two tailed, unequal variance) was used to determine the significance of the differences in the mean values ±SE (n = 24) of the CI between the two samples in the same developmental stage. P<0.05 was considered different. P<0.01 was considered significantly different.

### Analysis of soluble sugars and organic acids

The composition and concentrations of the soluble sugar and organic acid extracted from 3 g of frozen powdered pulp were determined by using an Agilent 6890N gas chromatograph (Agilent, USA) as described by Bartolozzi F et al. [34]. Three replicated extractions of each sample were performed. This experiment was performed twice within two years with similar results. Due to the similarity of the results, herein, the experiment data of one year is presented. Student's t test (two tailed, unequal variance) was used to determine the significance of the differences in the mean values ±SE (n = 3) of the content between the two samples in the same developmental stage. P<0.05 was considered different. P<0.01 was considered significantly different.

### RNA preparation for Illumina sequencing

The fruit pulps for each genotype at 166 DAA in 2010 were subjected to RNA-seq. The pulps of the samples, which were sampled from three different trees for each genotype, were mixed into a pool for RNA extraction. Approximate 2 to 3 g of powdered material was subjected to each RNA extraction, including 10 mL buffer extraction, 5 mL chloroform-isoamylalcohol extraction (twice), 5 mL isopropanol precipitation, 5 mL 75% ethanol cleaning and RNA purification as described by Camacho-Villasana YM et al. [35]. The total RNA was the mixture of five times of RNA extractions. An Agilent 2100 Bioanalyzer was used to determine the integrity and quality of the total RNA. The RNA with a RIN (RNA Integrity Number) value greater than 7 were considered qualified for RNA-seq. The beads with oligo(dT) were used to isolate poly(A) mRNA. The following procedures including RNA fragmentation, cDNA synthesis, size selection, PCR amplification and RNA-seq were performed at the Beijing Genome Institute (BGI) (Shenzhen, China). The obtained mRNA was fragmented into 200 nt to 700 nt by the fragmentation buffer (Ambion, Austin, TX). Then, random hexamer-primer was used to synthesize the first-strand cDNA using the cDNA Synthesis Kit (Stratagene, Cedar Creek, USA) following the manufacturer's protocol. The short fragments were purified using the QiaQuick PCR extraction kit (Qiagen, Valencia, CA) to repair the end by adding a poly(A) tail. Then, fifteen rounds of PCR amplification were carried out to enrich the purified cDNA. The cDNA library was sequenced using Illumina HiSeq 2000. Library quality control and quantification were performed with an Agilent 2100 Bioanalyzer and an ABI Step One Plus Real-Time PCR System.

### Illumina sequence analysis

25,629,358 and 25,801,572 clean reads for MT and WT were generated, representing 2,306,642,220 nt and 2,322,141,480 nt, respectively. The Q20 value (representing the accuracy of the sequencing) was greater than 94% for each sample. The following steps were used to filter the low-quality reads. 1) Remove the reads with adapters; 2) Remove the reads in which unknown bases were more than 10%; 3) Remove the low-quality reads (the percentage of low-quality bases was greater than 50% in a read; therefore, we defined a low-quality base as one whose sequencing quality was no greater than 10). After filtering, the remaining reads were called “clean reads” and used for downstream bioinformatic analysis. The mean length of the clean reads was approximately 90 nt with paired ends (Table 1). De novo transcriptome assembly was performed using a de Bruijn graph and the short reads assembling program (SOAPdenovo)with the default settings except for the K-mer value [36]. SOAPdenovo first combined reads with a certain length of overlap to form longer fragments without N, which were called contigs. Then, the reads were mapped back to the contigs. With paired-end reads, this program could detect contigs from the same transcript as well as the distances between these contigs. Next, SOAPdenovo connected the contigs using N to represent the unknown sequences between two contigs, thus a Scaffold was formed. The paired-end reads were used again for filling the gap between scaffolds to obtain sequences which had least Ns and could not be extended on either end. Such sequences were defined as unigenes, with average coverage of 89.28%and 79.77% for MT and WT, respectively. All of the unigenes that were identified by SOAPdenovo were subjected to a BLASTX alignment (e-value <0.00001) between unigenes and protein databases, including nr, Swiss-Prot, KEGG and COG. If the results of different databases conflicted with each other, a priority order of nr, Swiss-Prot, KEGG and COG was followed when determining the sequence direction of the unigenes. When a unigene could not be aligned with any of the above databases, ESTScan [37] was used to predict the coding regions and determine the sequence direction. The GO annotation of unigenes was performed using the Blast2GO program [38], and the GO functional classification of all the unigenes was performed so as to understand the distribution of gene functions of the species at the macro level using WEGO software [39]. To determine the functions of the gene products in metabolism processes and related cellular processes, KEGG Metabolic Pathway Analysis was performed.

**Table 1:** Summery of the transcriptome sequencing of ‘Jincheng’ (WT) and its mutant (MT).

	MT	WT	All
number of reads	25,629,358	25,801,572	51,430,930
total nucleotides(nt)	2,306,642,220	2,322,141,480	4,628,783,700
mean length of reads(nt)	90	90	
number of contigs	232,639	64,890	
mean length of contigs(nt)	161	250	
number of scaffolds	82,696	38,117	
mean length of scaffolds(nt)	312	376	
number of unigenes	57,547	24,034	44,413
mean length of unigenes(nt)	394	500	548

### Differential gene expression analysis

The gene expression level by RNA-seq was normalized by the reads per kb per million reads (RPKM) method [40]. The cutoff value to determine the gene transcriptional activity was determined based on a 95% confidence interval for all of the RPKM values of each gene.

The up or down regulation of a gene was decided according to its log2 (WT_RPKM/MT_RPKM) value. If log2 (WT_RPKM/MT_RPKM) >0, then a gene was up-regulated in WT. A GO Classification and KEGG Pathway Analysis of the differentially expressed unigenes (DEG) were performed to obtain an overall understanding of the transcriptome differences between the two samples.

DEGs with at least three database annotations were selected for further analysis. The proportions of up- and down-regulated unigenes of seven important pathways were analyzed to obtain a clear picture of the biological processes.

### Validation of RNA-seq data

The comparison between our data and the published data from csi.CDS.fa database was carried out by Blast 2.2.25 with an e-value cutoff of le-5.

To gain additional insight into the ripening-related processes, twenty-two genes that were involved in different fruit ripening processes were selected for validation by real-time quantitative RT-PCR (qRT-PCR). The total RNA of the fruit pulp mixture of samples representing per genotype per stage was extracted following the method described by Liu YZ et al. [41]. The sequences of the primer pairs (designed by Primer Express 3.0 (Applied Biosystems, Foster City, CA, USA)) were listed in Table 2. All of the qRT-PCRs were conducted using an ABI 7500 Real Time System (Applied Biosystems) with actin gene as the reference, following the method described by Keqin Yu et al. [22]. Two biological and three technical replications were performed.

**Table 2:** Candidate gene list and their primers for quantitative real time-PCR.

Name	Tamplate ID	Genome code of Citrus sinensis	Similarity (e-value)	Annotation	Primer sequence 5′ to 3′
**Abscisic acid metabolism and signal transduction pathway**					
CsNCED1	All-Unigene9577	Cs5g14370	0	9-cis-epoxycarotenoid dioxygenase [EC: 1.13.11.51]	Forward: AACCCGTCTGCCAGAACCTT
					Reverse: GTTGGCTCCGTTTCTGACGTA
CsNCED3	All-Unigene111	Cs6g19500	3.58E-07	9-cis-epoxycarotenoid dioxygenase [EC: 1.13.11.51]	Forward: GCTTCCGTTTGTGGCCTACTT
					Reverse: ATTGACCCGGCATTTTTATGTG
CsAAO	All-Unigene40211	Cs8g13770	7.76E-15	abscisic-aldehyde oxidase [EC: 1.2.3.14]	Forward: CGCATGCGTTGTCCTACTGT
					Reverse: AAGACCTTCGCTTGTGGTAATTG
CsABA8ox1	All-Unigene16990	Cs6g19380	5.00E-132	()-abscisic acid 8′-hydroxylase [EC: 1.14.13.93]	Forward: GCCCAAAAGTCAAAGGACAAGT
					Reverse: CATCATCATTTCGGCTTTCCA
CsABA8ox3	All-Unigene8405	Cs1g09250	4.03E-03	()-abscisic acid 8′-hydroxylase [EC: 1.14.13.93]	Forward: TTAAGAATGGAACCGCCGAAT
					Reverse: TTGGGAATGGTGTATCCATCAA
CsABI1	All-Unigene41252	Cs4g20430	0	ABA-insensitive 1; K01090 protein phosphatase [EC: 3.1.3.16]	Forward: GCCTCCTCCAAACTTGATTGC
					Reverse: CCCTCAAACCCTCAGCAGAA
CsAHG1	All-Unigene1776	Cs9g16360.1	1.36E-02	ABA-Hypersensitive germination1; K01090 protein phosphatase [EC: 3.1.3.16]	Forward: GGGCCTCGGATGGTAGAAGA
					Reverse: AGCAAGCCGGGTTAACAATG
CsAHG3	All-Unigene15798	Cs7g31880.1	9.04E-11	ABA-Hypersensitive germination3	Forward: GCTAGAGCTCCGTCCGTTTAAC
					Reverse: GCTTCTTTCGTTTCCGATCGT
CsHAB1	All-Unigene40938	Cs1g17890.9	5.73E-03	Hypersensitive to ABA1; K14497 protein phosphatase 2C [EC: 3.1.3.16]	Forward: GGCAAGGTCATCCAATGGAA
					Reverse: AGCAGAGAAATAACACAATGTCAAAGA
CsHAB2	All-Unigene40938	Cs1g17890.4	5.73E-03	Hypersensitive to ABA2; K14497 protein phosphatase 2C [EC: 3.1.3.16]	Forward: CACTGTGCAATGCCTAGTCAGTATAC
					Reverse: TGTGGCGGCAGACAGTCAT
CsHAI1	All-Unigene8244	Cs8g19140.1	3.10E-06	Highly ABA-Induced1	Forward: CACGTTTGTCCCCACCAGAT
					Reverse: CGTTGGCTTGCTGCTGCTA
CsPYL4	All-Unigene756	Cs7g30500.1	3.18E-123	PYR1-like protein 4; K14496 abscisic acid receptor PYR/PYL family	Forward: GGAAACTTGCACTTTTGTTGAGACT
					Reverse: TAGCAGCCATGTTCTCCGAAA

Name	Tamplate ID	Genome code of Citrus sinensis	Similarity (e-value)	Annotation	Primer sequence 5′ to 3′
CsPYL8	All-Unigene4394	Cs5g02360.2	0	PYR1-like protein 8; K14496 abscisic acid receptor PYR/PYL family	Forward: TGGCAAGACTGAGGAGGACTACA
					Reverse: TAACAAGCGTAGAGCTGCACTGA
CsPYL9	All-Unigene4394	Cs5g02360	0	PYR1-like protein 9; K14496 abscisic acid receptor PYR/PYL family	Forward: GGAAAACAAGGCCACTAACATGA
					Reverse: GTATCCTTCTTCTTGTCACAGTCCAA
CsPYR1	All-Unigene5668	orange1.1t01026	0	Pyrabactin resistance 1; K14496 abscisic acid receptor PYR/PYL family	Forward: GTCACCTCGGTGCATGGATT
					Reverse: TCGGGCACATCAACAACGTA
CsSnRK2.2	All-Unigene1517	Cs1g23060.1	2.17E-36	Suc nonfermenting-related kinase group 2; K08286 protein-serine/threonine kinase [EC: 2.7.11.-]	Forward: TGTTCACCAGAATCCGATACCA
					Reverse: GCTGACACGACACCGTTTTAGA
**Sucrose metabolism and signal transduction pathway**					
CsSUS	All-Unigene4586	Cs4g06850	0	sucrose synthase [EC: 2.4.1.13]	Forward: CATGGCCTCAACAACAGTCAA
					Reverse: AAGGGAGCTTTTGTGCAACCT
CsSPS	All-Unigene14046	Cs7g05690	5.00E-110	K00696 sucrose-phosphate synthase [EC: 2.4.1.14]	Forward: TTGATGAAGCGTGTGACAGCTA
					Reverse: CTGCCCCTTGAGTACCCCTAA
CsSUC1	All-Unigene6487	Cs3g22560	0	citrus sucrose transporter 1 [Citrus sinensis]	Forward: TCTTCATGGCCGTCGGTAAC
					Reverse: CGGAAGCATGTGGTACAAGTGA
CsSUC3	All-Unigene219	Cs7g04100	8.38E-07	sucrose transporter 3	Forward: CATCACCCGTCTCAAGTTCGA
					Reverse: AGTTTGGCCGGGCTAAGG
CsSUC4	All-Unigene7099	Cs3g16640	5.26E-49	sucrose transporter 4	Forward: GCCGTCGCAGACCGTTTAT
					Reverse: GCGGAAAGACCAATTAGCAAA
**Jasmonic acid metabolism and signal transduction pathway**					
CsLOX	All-Unigene25245	Cs3g13930	9.00E-12	K00454 lipoxygenase [EC: 1.13.11.12]	Forward: TCATGCCCGAACCAGGAA
					Reverse: GAAGGCCAAGTCAGGGTTCTT
CsAOS	All-Unigene15147	Cs3g24230.1	2.24E-08	allene oxide synthase [Citrus sinensis]	Forward: CTTCTGCCAACCGAATTTCAA
					Reverse: CGAACCCGAGGAGTGTATAGCT
CsJMT	All-Unigene37066	Cs6g18060.1	0	K08241 jasmonate O-methyltransferase [EC: 2.1.1.141]	Forward: AATTCTTGTGGTGTTGGGAAGTAAC
					Reverse: CAGGGATTGCTGAGTGAAGCT
CsCOI1	All-Unigene15083	Cs3g17560.1	3.54E-05	coronatine insensitive 1	Forward: TGCCGCTCCTTCTATCAACA
					Reverse: GGACCCGGAAACAGCTTCTC

Name	Tamplate ID	Genome code of Citrus sinensis	Similarity (e-value)	Annotation	Primer sequence 5′ to 3′
CsJAZ3	All-Unigene11744	Cs2g17230.1	0	JAZ3 (Jasmonate-ZIM-domain protein 3); K13464 jasmonate ZIM domain-containing protein	Forward: TTGCTGCAACTGCTGTGACA
					Reverse: CTGAGGGATGCCCTTGTAAAAG
CsJAZ8	All-Unigene2433	Cs2g03240.2	4.00E-146	JAZ8 (Jasmonate-ZIM-domain protein 8)	Forward: GCGTGAGCCCGCAAAAT
					Reverse: TGCAAACTTGTCCGTTGTAGAAGA
**Other fruit -ripening related genes**					
CsPSY	All-Unigene40939	Cs6g15910.4	9.52E-11	K02291 phytoene synthase [EC: 2.5.1.32]	Forward: TGTTGGTCAGGTACAGGTAAGATCA
					Reverse: CAATCGCTCACCCCATGAAC
CsPME	All-Unigene12810	Cs5g09370.1	0	K01051 pectinesterase [EC: 3.1.1.11]	Forward: ATTTGCCATTCCAGCCAAGT
					Reverse: GCTAAGCTTTCCCTCGTCTCACT
CsZDS	All-Unigene25452	orange1.1t02361	0	K00514 zeta-carotene desaturase [EC: 1.14.99.30]	Forward: CTTCCAGCTCCGCTAAATGG
					Reverse: CGGCCAAGTCAGCATTTCAT

A liner regression analysis and correlation coefficient calculations were made between the RNA-seq and qRT-PCR data at the same stage using Excel 2003. The RNA-seq data were first returned to 2^×^.

### Expression analysis of candidate genes

The twenty-seven genes involved in the metabolism and signal transduction pathways of abscisic acid, sucrose and jasmonic acid and three other generally acknowledged fruit ripening related genes were subjected to qRT-PCR to form an expression profile of the 6 fruit ripening stages from 139 DAA to 232 DAA. The procedure was performed as above. The results were analyzed using Cluster 3.0.

## Results

### Fruit quality analysis

**Figure 1:**
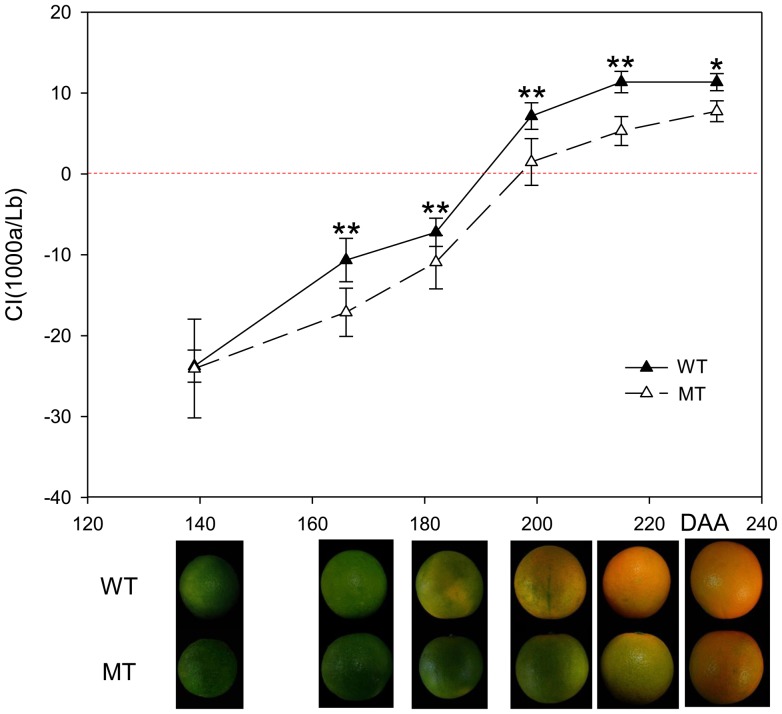
Changes in the peel color of the wild type of ‘Jincheng’ sweet orange (WT) and its mutant (MT) fruit during fruit development and ripening. The data represent the mean values with twenty-four replicates. The asterisks indicate values that were determined by Student's t test to be different (P<0.05) between the two samples. Double asterisks indicate significant differences (P<0.01). DAA, days after anthesis; the upper fruits were WT; the lower fruits were MT.

**Figure 2:**
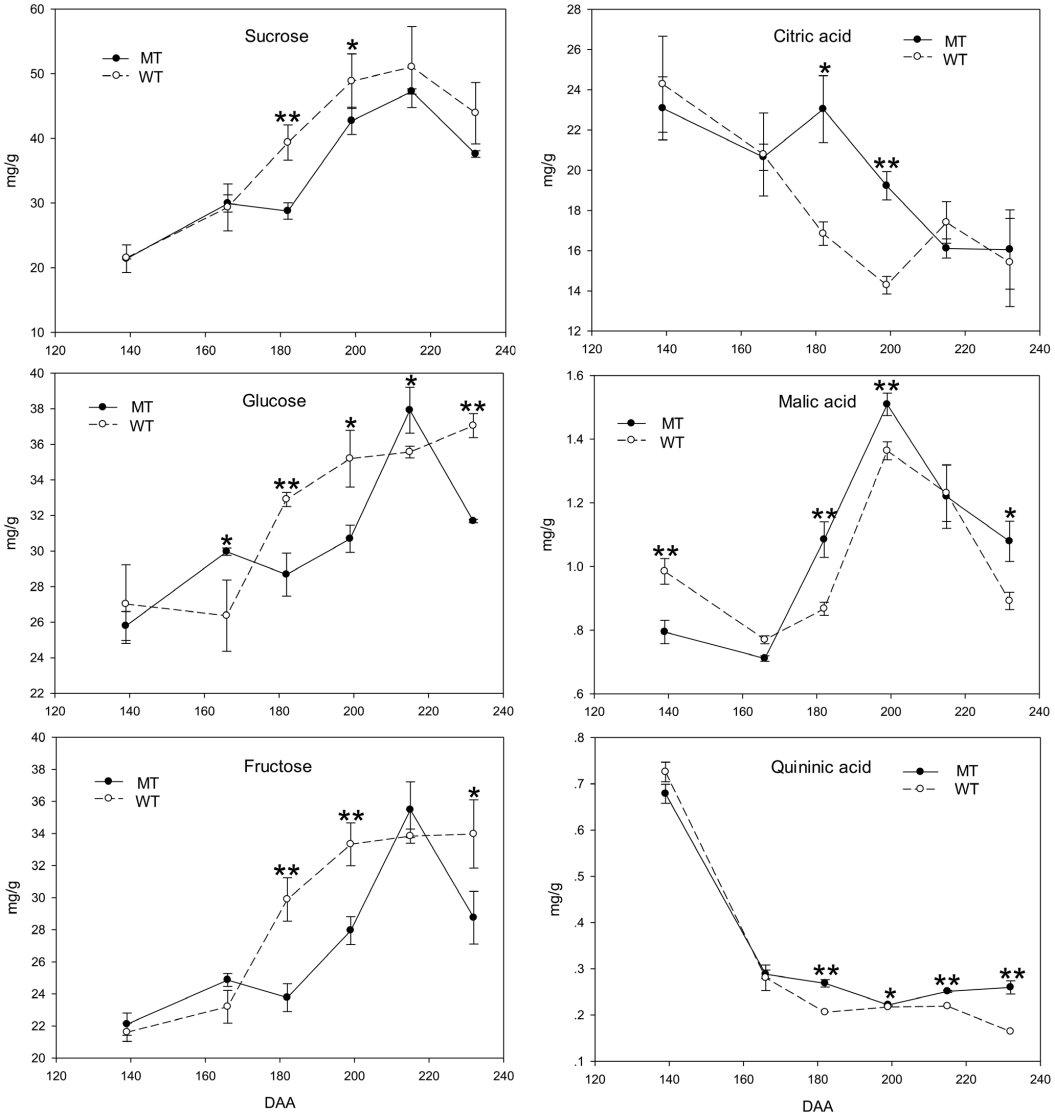
Changes in the soluble sugar and acid contents of the fruit flesh of the wild type of ‘Jincheng’ sweet orange (WT) and its mutant (MT) during fruit development and ripening. The data represent the mean values with at least three replicates. The asterisks indicate values that were determined by Student's t test to be different (P<0.05) between the two samples. Double asterisks indicate significant differences (P<0.01).

The dynamic changes in the fruit color, soluble sugar content and organic acid content were analyzed in the wild type ‘Jincheng’ sweet orange (WT) and its late-ripening mutant (MT) during six ripening stages. Compared with WT, there was a delayed color-break in MT starting at 166 DAA according to the fruit pictures and the color index (CI) data (Fig. 1). Fig. 2 demonstrated that the contents of all the three sugars increased throughout fruit development, while all the three acids showed an opposite trend. The differences between MT and WT in the contents of most soluble sugars and acids began to arise from 182 DAA, except for glucose from 166 DAA. The total soluble sugar content was lower in MT than in WT during the fruit developing and ripening processes. However, the total organic acid content was higher in MT than in WT from 166 DAA to 215 DAA according to the gas chromatograph data (Fig. 2).

### Illumina sequencing and reads assembly

To characterize the whole transcriptome differences between the two samples in the early fruit ripening stage, the fruits at 166 DAA were used for transcriptional analysis using Illumina HiSeq 2000.

25,629,358 and 25,801,572 clean reads representing 2,306,642,220 and 2,322,141,480 nucleotides were generated for MT and WT, respectively. The mean length of the reads was approximate 90 nt, encompassing 2 Gb of sequence data for each sample. 232,639 and 64,890 contigs with their corresponding mean lengths of 161 nt and 250 nt were generated for MT and WT, respectively. In addition, 82,696 and 38,117 scaffolds, with their corresponding mean lengths of 312 nt and 376 nt, respectively, were assembled for each genotype. Ultimately, 57,547 and 24,034 unigenes, with average lengths of 394 nt and 500 nt, were gained respectively for MT and WT. Altogether, 44,413 unigenes were generated, ranging from 200 nt to 6,116 nt, including 5,827 unigenes (13.12% of all of the unigenes) that were larger than 1000 nt (Table 1).

### Analysis of all unigenes

**Figure 3:**
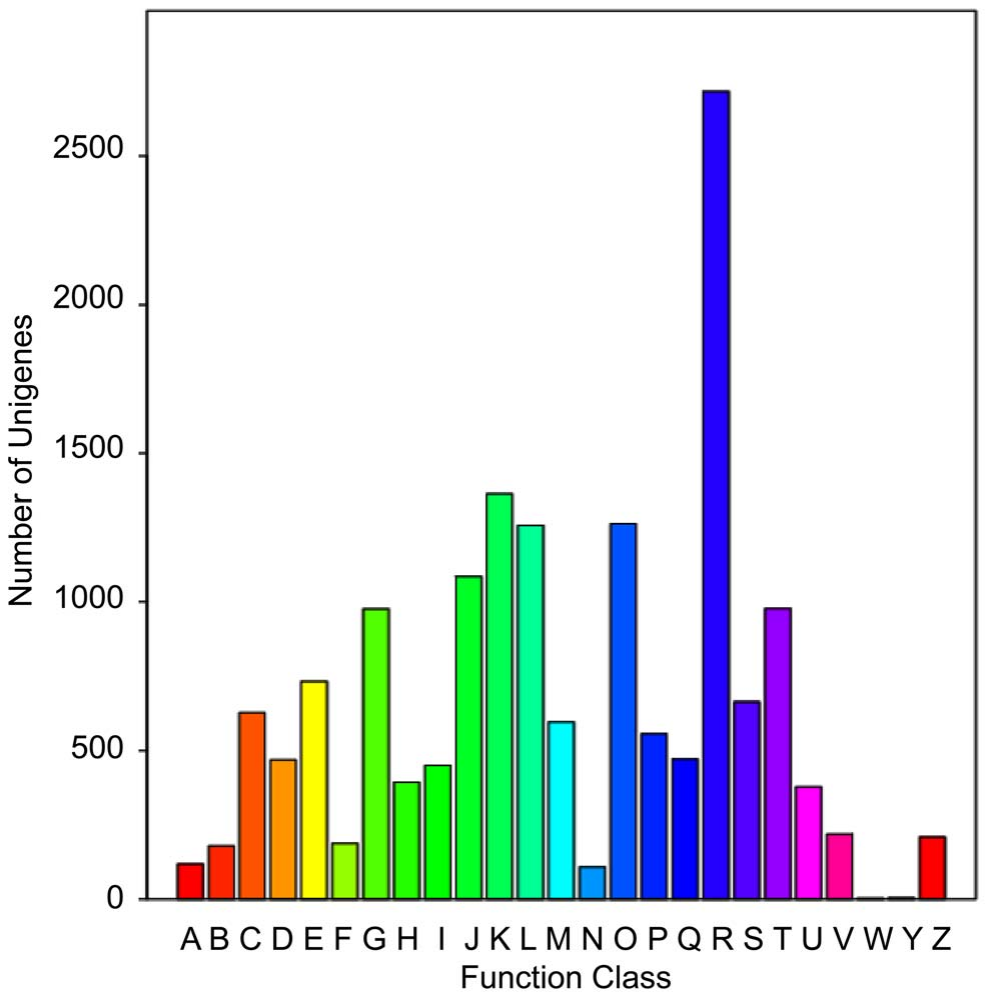
Histogram presentation of clusters of orthologous groups (COG) classification. Out of 44,413 unigenes, 9386 sequences have a COG classification among the 25 categories. A. RNA processing and modification; B. Chromatin structure and dynamics; C. Energy production and conversion; D. Cell cycle control, cell division, chromosome partitioning; E Amino acid transport and metabolism; F. Nucleotide transport and metabolism; G. Carbohydrate transport and metabolism; H. Coenzyme transport and metabolism; I. Lipid transport and metabolism; J. Translation, ribosomal structure and biogenesis; K. Transcription; L. Replication, recombination and repair; M. Cell wall/membrane/envelope biogenesis; N. Cell motility; O. Posttranslational modification, protein turnover, chaperones; P. Inorganic ion transport and metabolism; Q. Secondary metabolites biosynthesis, transport and catabolism; R. General function prediction only; S. Function unknown; T. Signal transduction mechanisms; U. Intracellular trafficking, secretion, and vesicular transport; V. Defense mechanisms; W. Extracellular structures; Y. Nuclear structure; Z. Cytoskeleton.

All of the unigenes were annotated using the Nr, Swiss-Prot, KEGG, and COG databases. Among these unigenes, 31,368 unigenes (70.63% of the total) were matched with at least one database. Then the COG and GO classifications were performed, followed by a KEGG pathway analysis. In the COG classification, the top five enriched categories were K (Transcription, 8.52%), O (Posttranslational modification, protein turnover, chaperones, 7.89%), L (Replication, recombination and repair, 7.86%), J (Translation, ribosomal structure and biogenesis, 6.79%) and T (Signal transduction mechanisms, 6.11%) (Fig. 3 and S1 Table). In the GO classification, the top five clustered classes in function were binding (43.02%), catalytic activity (40.50%), transporter activity (5.67%), transcription regulator activity (2.82%) and structural molecule activity (2.68%) (S1 Table). In the KEGG pathway analysis, the top five clustered classes were metabolic pathways (23.34%), plant-pathogen interaction (7.46%), spliceosome (5.61%), biosynthesis of plant hormones (4.72%), and biosynthesis of phenylpropanoids (4.19%) (S1 Table).

### Analysis of all of the differentially expressed genes (DEGs)

**Figure 4:**
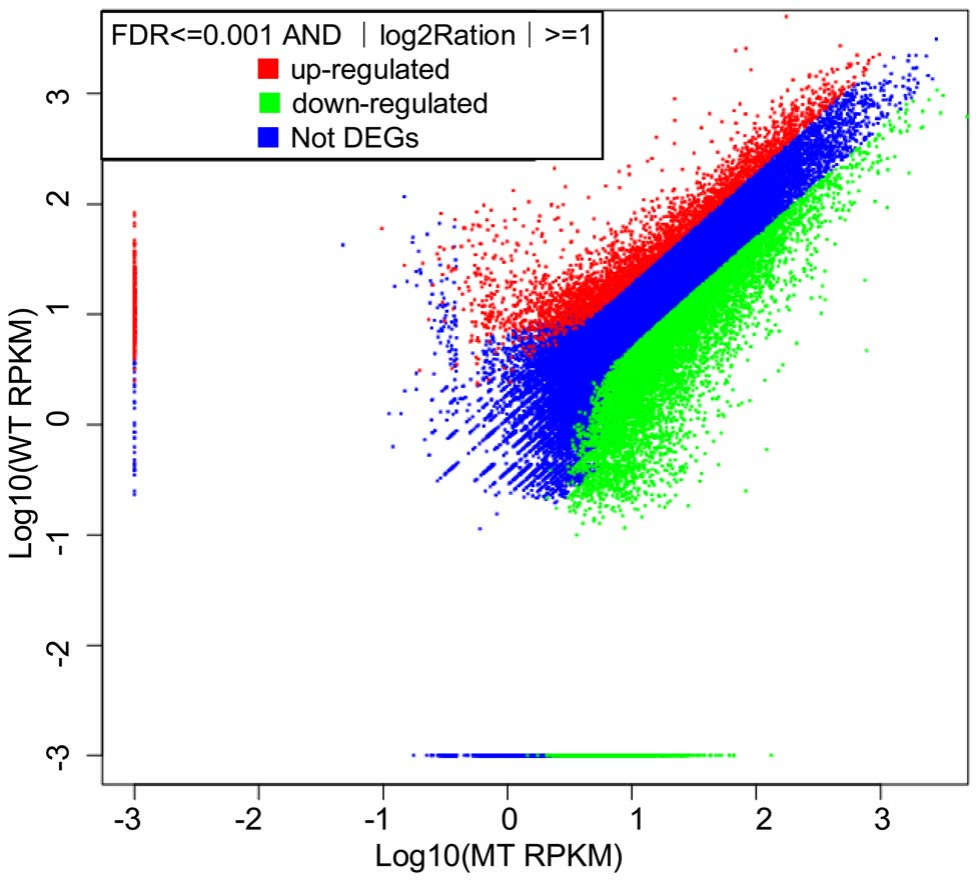
The distribution of MT-vs-WT DEGs. There were nearly three times more down-regulated genes than up-regulated genes. WT. the wild type of ‘Jincheng’ sweet orange; MT. the late-ripening mutant of ‘Jincheng’ sweet orange.

Of all unigenes, 13,412 unigenes with a false discovery rate (FDR) ≤−0.001 and |log2Ratio| ≥−1 were identified as DEGs, accounting for 30.20% of all unigenes (S2 Table). Of the DEGs, 24.2% were up-regulated in WT, while 75.8% were down-regulated (Fig. 4). According to the GO classification, the top five clustered classes in function were binding (42.59%), catalytic activity (39.58%), transporter activity (6.10%), structural molecule activity (3.55%) and electron carrier activity (2.62%). In the KEGG pathway analysis, the top five clustered classes were metabolic pathways (22.79%), plant-pathogen interaction (7.10%), spliceosome (7.02%), biosynthesis of plant hormones (4.09%) and biosynthesis of phenylpropanoids (3.65%) (S3 Table).

**Figure 5:**
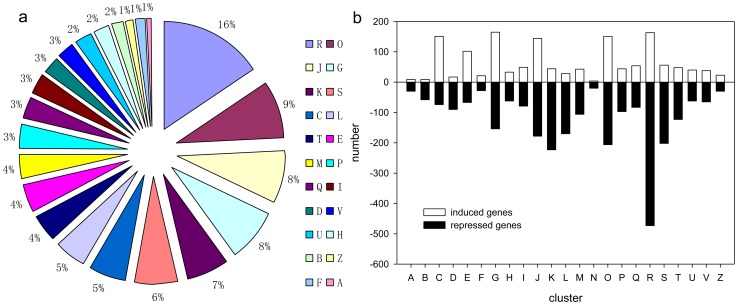
The classification of all DEGs with annotations from at least three databases: (a) the annotation according to the Nr, COG, GO and KEGG databases; (b) the distribution of up (in white) and down-regulated (in black) unigenes in the wild type of ‘Jincheng’ sweet orange for each cluster. Cluster A. RNA processing and modification; B. Chromatin structure and dynamics; C. Energy production and conversion; D. Cell cycle control, cell division, chromosome partitioning; E. Amino acid transport and metabolism; F. Nucleotide transport and metabolism; G. Carbohydrate transport and metabolism; H. Coenzyme transport and metabolism; I. Lipid transport and metabolism; J. Translation, ribosomal structure and biogenesis; K. Transcription; L. Replication, recombination and repair; M. Cell wall/membrane/envelope biogenesis; N. Cell motility; O. Posttranslational modification, protein turnover, chaperones; P. Inorganic ion transport and metabolism; Q. Secondary metabolites biosynthesis, transport and catabolism; R. General function prediction only; S. Function unknown; T. Signal transduction mechanisms; U. Intracellular trafficking, secretion, and vesicular transport; V. Defense mechanisms; Z. Cytoskeleton.

Among the DEGs, 4116 unigenes (30.69% of all of the DEGs) were annotated by at least three different databases. These unigenes could be categorized into 23 clusters (Fig. 5; S4 Table). The top five enriched categories were respectively O (Posttranslational modification, protein turnover, chaperones, 8.67%), J (Translation, ribosomal structure and biogenesis, 7.82%), G (Carbohydrate transport and metabolism, 7.75%), K (Transcription, 6.49%) and C (Energy production and conversion, 5.47%).

To further understand the transcriptional differences between MT and WT, more specific sub-classifications were performed for the significantly enriched five categories, along with fruit development-related categories, such as signal transduction (T) and secondary metabolism mechanism (Q) (S1–S7 Figs.). In the posttranslational modification subcategory (O), chaperones and folding catalysts (38%) and ubiquitin system (25%) were the two largest groups with more than half of the genes down-regulated, indicating the importance of these two pathways in fruit ripening (S1 Fig.). In the translation subcategory (J), the top three groups were ribosome (46%), translation factors (14%) and spliceosome (8%). Most genes of these 3 groups were down-regulated, except for the ribosome category (S2 Fig.). In the subcategory of carbohydrate transport and metabolism (G), starch and sucrose metabolism (17%), glycolysis/gluconeogenesis (16%) and glycan biosynthesis and metabolism (13%) were the notably enriched groups with the number of up-regulated genes similar to that of down-regulated genes (S3 Fig.). In the transcription subcategory (K), the largest groups were transcription factors (39%), replication and repair (16%), and spliceosome (12%) (S4 Fig.). In the subcategory of energy production and conversion (C), energy metabolism (60%), carbohydrate metabolism (14%), and amino acid metabolism (9%) were the three largest groups (S5 Fig.). In the subcategory of signal transduction mechanism (T), the top three remarkably enriched groups were cell growth and death (11%), circadian rhythm–plant (10%), and plant-pathogen interaction (9%) (S6 Fig.). In the subcategory of secondary metabolites biosynthesis, transport and catabolism (Q), metabolism of terpenoids and polyketides (45%), flavonoid biosynthesis (12%), and phenylpropanoid biosynthesis (11%) were the most abundant groups (S7 Fig.). In total, the proportion of the down-regulated DEGs in WT was respectively 83.52% for K, 71.93% for T, 60.58% for Q, 57.98% for O, 55.28% for J, 48.28% for G, and 30.98 for C. These results suggest that during the early ripening stage, the lower the ripening degree was, the weaker the overall energy metabolism activity was (S5 Fig.), but the higher the overall transcription activity (S4 Fig.) and signal transduction (S6 Fig.) abilities were. There were no difference in the overall carbohydrate metabolism (S3 Fig.) and translation activities (S2 Fig.) between the two ripening degrees (WT and MT); it may possibly be due to that metabolism and translation are the most basic biologic processes of plant.

### Validation of the RNA-seq data

**Figure 6:**
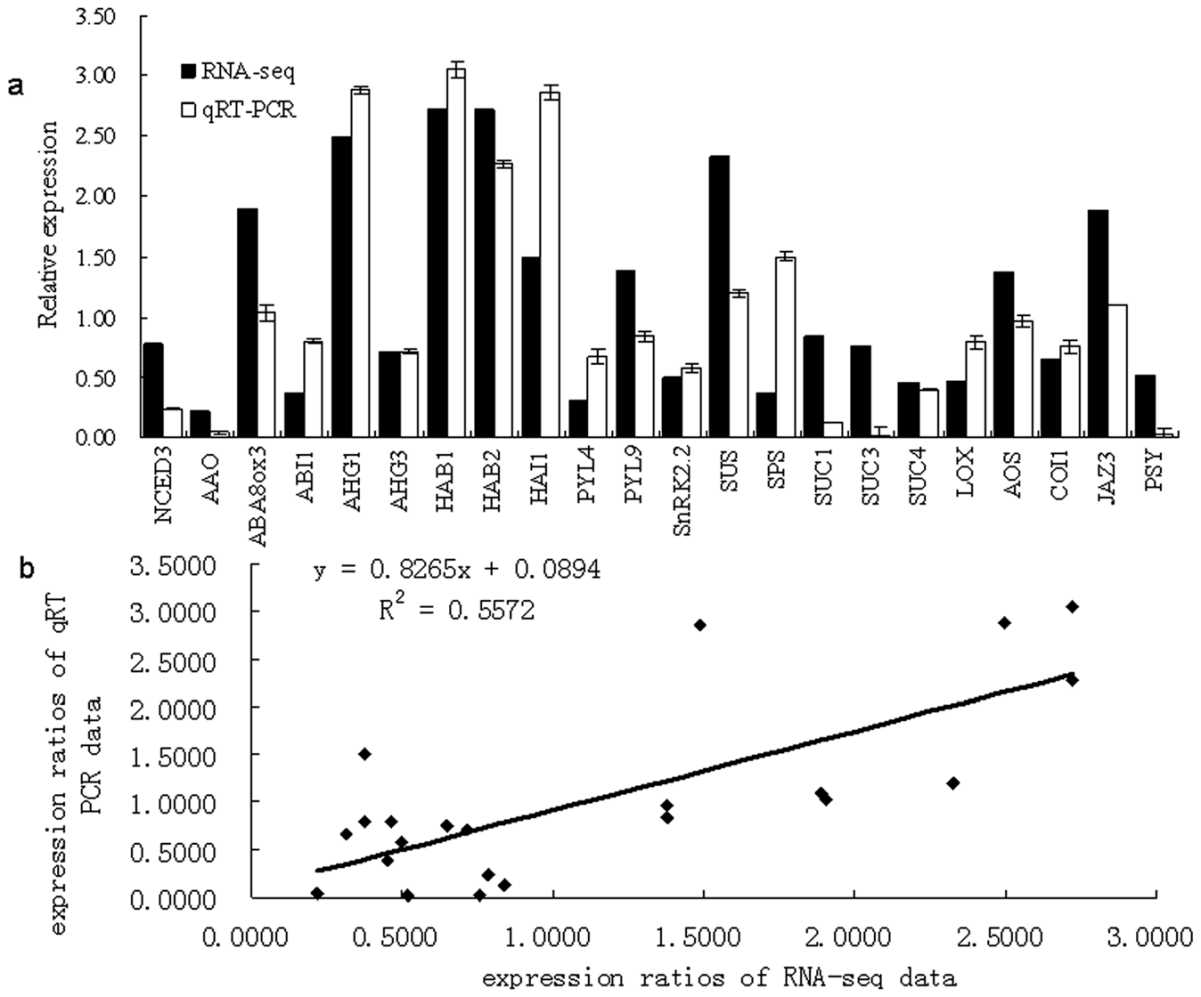
Comparison of gene expression ratios that were obtained by RNA-seq and by quantitative real-time PCR (qRT-PCR): (a) the comparison of the gene expression value gained by RNA-seq and by qRT-PCR; (b) the liner regression analysis between the gene expression ratios obtained by RNA-seq and by qRT-PCR. The black bars represent RNA-seq data, and the white bars represent the qRT-PCR data. The RNA-seq data were first restituted to 2^×^. The linear regression [(RNA-seq value)  =  a (RT-PCR value) + b] analysis indicated a positive relationship between them.

Since Qiang Xu et al [42] and Fred G. Gmitter et al [43] have published the genome sequences of *Citrus sinensis*, we compared our RNA-seq data with the data from their CDS databases using Blast 2.2.25 with an e-value cutoff of le-5. 35,497 unigenes (79.92%) and 33,910 unigenes (76.35%) respectively matched to the *C.sinensis* CDS databases from Xu and Gmitter (S5 Table and S6 Table). Furthermore, there were 18,100 unigenes (40.75%) and 31,027 unigenes (69.86% of all) whose sequence similarities reached 100% with the data from Xu's database and Gmitter's, respectively. In addition, 33,776 unigenes (76.05%) and 32,156 unigenes (72.39%) had sequence similarities of greater than 95%, respectively. Thus the reliability of the RNA-seq data was confirmed. Moreover, the RNA-seq provided new sequence information for the citrus transcriptome. Twenty-two genes with various degrees of expression levels were subjected to quantitative real time-PCR (qRT-PCR) to further validate the RNA-seq data (Fig. 6a). The primer sequences and detailed information are shown in Table 2. The linear regression [(RNA-seq value)  =  a (RT-PCR value) + b] analysis showed a correlation coefficient of 0.75 indicating a positive correlation between the RNA-seq data and the qRT-PCR data (Fig. 6b).

### Expression analysis of the candidate genes and pathways

According to the qRT-PCR results, we found that during this early ripening stage, the expression levels of the sucrose biosynthesis genes were higher than that of the sucrose transporter genes. While the expression levels of the abscisic acid (ABA) biosynthesis genes were lower than that of the PP2C genes. Moreover, the expression levels of the genes in the jasmonic acid (JA) biosynthesis and signal transduction pathways were at moderate level (Fig. 6). To gain more detailed information on the three pathways, we analyzed the expression profile throughout the entire fruit ripening stages. Thirty genes were chosen, including twenty-seven key genes involved in the three pathways and three generally acknowledged ripening-related genes, namely *pectinesterase* (*PME*), *phytoene synthase* (*PSY)*, *and zeta-carotene desaturase* (*ZDS*) (Table 2).

**Figure 7:**
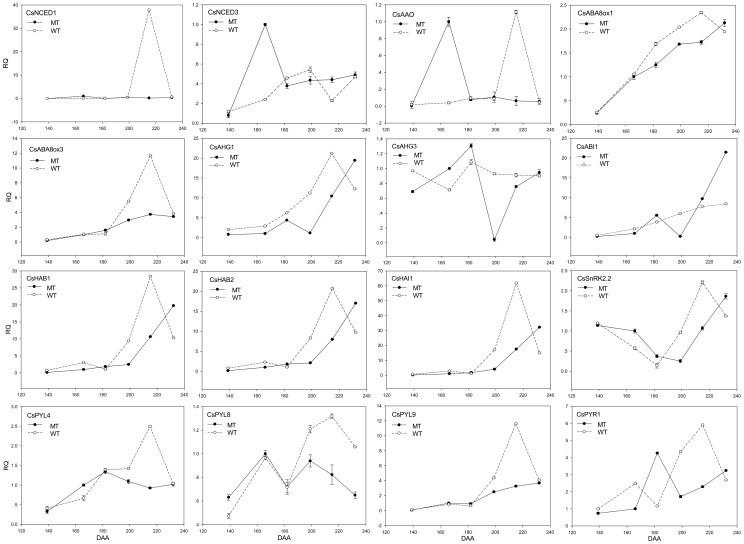
Transcript levels of the genes in the ABA metabolism and signal transduction pathways in the wild type of ‘Jincheng’ sweet orange (WT) and its late ripening mutant (MT) during fruit development and ripening. *Actin* was used as the internal control. The error bars represent SE (n = 3). NCED, 9-cis-epoxycarotenoid dioxygenase; AAO, ABA-aldehyde oxidase; ABA8ox1, ABA 8′-hydroxylase 1; ABA8ox3, ABA 8′-hydroxylase 3; AHG1, ABA-Hypersensitive germination1; AHG3, ABA-Hypersensitive germination3; ABI1, ABA insensitive 1; HAB1, Hypersensitive to ABA1; HAB2, Hypersensitive to ABA2; HAI1, Highly ABA-Induced1; PYL2, 4, 8, 9, PYR1-like proteins; PYR1, Pyrabactin resistance 1; SnRK2, Suc non-fermenting-related kinase group 2.

**Figure 8:**
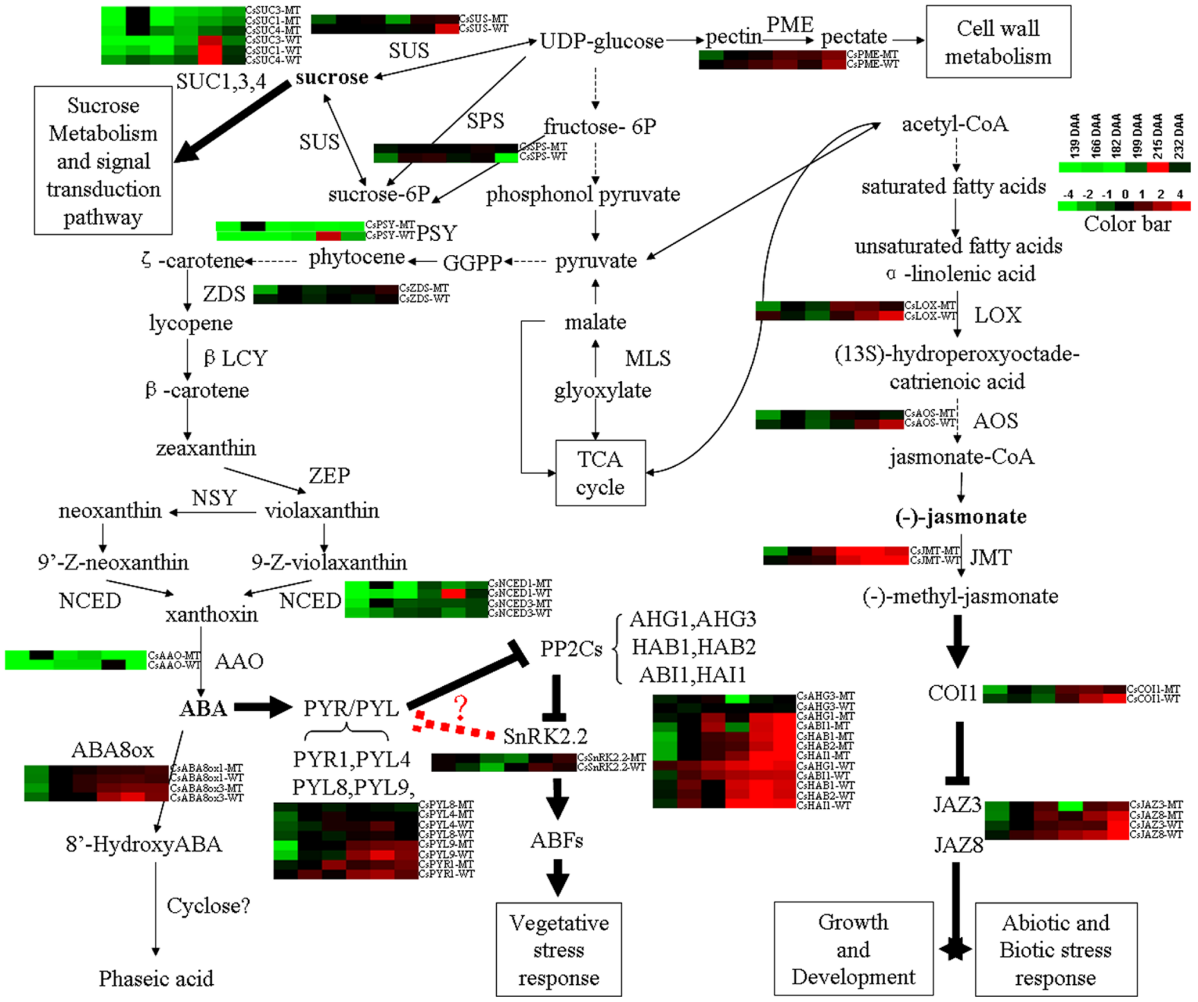
Summary of the possible relationship of all the candidate genes in the fruit ripening regulatory mechanism. The heat maps showed the expression level of the genes nearby. SPS, sucrose-phosphate synthase; SUS, sucrose synthase; SUC, sucrose transporter; PME, pectinesterase; PSY, phytoene synthase; ZDS, zeta-carotene desaturase; MLS, malate synthase; βLCY, lycopene β-cyclase; ZEP, zeaxanthin epoxidase; NSY, neoxanthin synthase; NCED, nine-cis-epoxycarotenoid dioxygenase; AAO, abscisic-aldehyde oxidase; ABA8ox, abscisic acid 8′-hydroxylase; AHG, ABA-Hypersensitive germination; HAB, Hypersensitive to ABA1; ABI1, *ABA insensitive 1*; HAI1, Highly ABA-Induced1; PYR1, Pyrabactin resistance 1; PYL, PYR1-like protein; SnRK2.2, Suc nonfermenting-related kinase group 2; ABFs, Abscisic acid response element Binding Factors; LOX, lipoxygenase; AOS, allene oxide synthase; JMT, jasmonate O-methyltransferase; COI1, coronatine insensitive 1; JAZ, Jasmonate-ZIM-domain protein.

**Figure 9:**
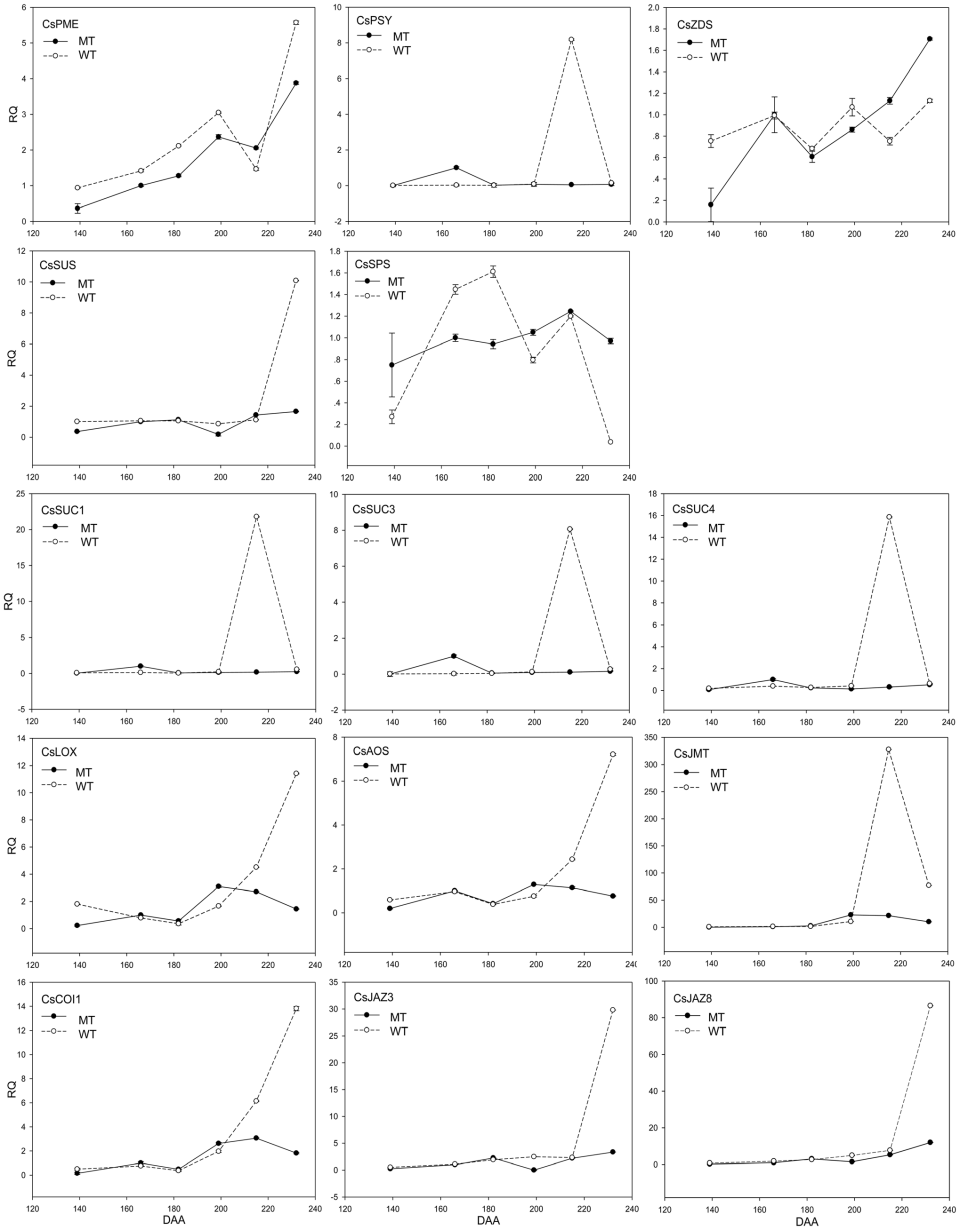
Transcript levels of the *PSY* gene, and the genes in the sucrose and jasmonic acid metabolism and signal transduction pathways in the wild type of ‘Jincheng’ sweet orange (WT) and its late ripening mutant (MT) during fruit development and ripening. *Actin* was used as the internal control. The error bars represent SE (n = 3). PSY, Phytoene synthase; SPS, Sucrose phosphate synthase; SUS, Sucrose synthase; SUC1, 3, 4, Sucrose transporters ; LOX, Lipoxygenase; AOS, Allene oxide synthase; JMT, Jasmonate O-methyltransferase; COI1, coronatine insensitive 1; JAZ, Jasmonate-ZIM-domain protein.

The expression profiles of the genes in the ABA metabolism and signal transduction pathways are shown in Fig. 7 and Fig. 8. The 9-cis-epoxycarotenoid dioxygenase (*CsNCED1* and *CsNCED3*) gene and the abscisic-aldehyde oxidase (*CsAAO*) gene are the key genes in the ABA biosynthesis pathway. Their expression levels peaked at 182 DAA in MT. However, the highest expression of *CsNCED1* and *CsAAO* was at 215 DAA in WT, and the highest expression of *CsNCED3* was at 199 DAA in WT. In addition, *CsNCED1* might play a leading role because its expression level was much higher than that of the other two genes. The expression level of abscisic acid 8′-hydroxylase (*CsABA8ox*) boosted with the fruit ripening degree, so did *CsPME* (Fig. 9), suggesting that *PME* can be indicate the fruit ripening degree. The expression levels of most the PP2C genes gradually increased in both MT and WT, but the increase in MT came later. While ABA-hypersensitive germination3 (*CsAHG3*) gene exhibited an irregular trend. The PYR/PYL family genes exhibited similar expression trend during the fruit ripening process. What's more, *CsPYL9* had the highest expression level among all of the *PYL* members. The expression trend of *CsPYL4* was the most similar to that of *CsPYR1* suggesting that the interaction between *CsPYL4* and *CsPYR1* was strong in citrus. The curve of the expression trend of Suc non-fermenting-related kinase group 2 (*CsSnRK2.2*) looked like a “V” (It fell first, and then rose). In MT, the expression level of *CsSnRK2.2* was lowest at 199 DAA. In WT, the expression level of *CsSnRK2.2* was lowest at 182 DAA. After that day, it increased and peaked at 215 DAA and then declined. Overall, the expression level of the ABA -receptors and -responsive genes increased gradually during the citrus fruit ripening process.

The expression profiles of the genes in the sucrose metabolism and signal transduction pathways are shown in Fig. 9 and Fig. 8. The expression trends of sucrose synthase (*CsSUS*) and sucrose-phosphate synthase (*CsSPS*) were different during the fruit ripening process. During the first two stages, the expression level of *CsSUS* was low in both WT and MT, so was the sucrose content (Fig. 2). It was possibly because the activity of metabolism was very strong and the photosynthetic products were consumed. At 166 DAA and 182 DAA, the expression level of *CsSPS* increased quickly and was much higher in WT than in MT. Therefore, the sucrose content became higher in WT than in MT from 182 DAA. Sucrose began to accumulate in the fruits. During the final stage, the expression level of *CsSUS* in WT increased suddenly, while its level in MT remained. This trend led to a higher content of glucose and fructose in WT than in MT at 232 DAA, while the content of sucrose decreased. During this stage, the citrus fruit was completely ripe, and hexose accumulated quickly. The expression level of Cs*SUC* was much higher in WT than in MT during the final ripening stages, suggesting that the sucrose in WT fruits was enough to trigger the down-stream metabolic processes. The expression trend of Cs*SUC* was consistent with that of phytoene synthase (*CsPSY*) (Fig. 9). These results indicated that *CsSPS* was closely related to the increase of sucrose in fruits after color-break, and that *CsSUS* mainly functions during the late ripening stage of fruit (Fig. 2 and Fig. 9).

The expression profiles of the genes in the jasmonic acid (JA) metabolism and signal transduction pathways are also shown in Fig. 9 and Fig. 8. The JA biosynthesis genes lipoxygenase (*CsLOX*) and allene oxide synthase (*CsAOS*) shared a similar expression pattern. The expression levels of these genes were lower in MT than in WT during the first and last two stages. The overall trends of *CsLOX and CsAOS* were slightly rising in MT and it reached peak at 182 DAA in WT. Jasmonate O-methyltransferase (*JMT*) was a key enzyme in the JA-regulated plant response processes, and the expression level of *JMT* was significantly higher than that of *CsLOX* and *CsAOS*, especially during the late ripening stages. *JMT*'s expression level was significantly higher in WT than in MT during the last two ripening stages. The expression pattern of coronatine insensitive 1 (*CsCOI1*) was close to that of *CsLOX* and *CsAOS*. The expression level of the Jasmonate-ZIM-domain protein (*CsJAZ*) was low at the beginning and was on a slow rise. However, after 215 DAA, it began to increase rapidly, and it was especially true with WT. The different gene expression patterns in JA metabolism and signal transduction pathways between MT and WT might contribute to the differences in the fruit ripening time between MT and WT.

The relationship between these three pathways is shown in Fig. 8. From a macro point of view, during citrus fruit ripening process, the sucrose biosynthesis and cell wall degradation function all the way. However, plant hormones, mainly ABA and JA function in the late ripening process. In addition, genes in the signal transduction pathway have much higher expression levels than genes in the biosynthesis pathway. The different transcription levels of these thirty genes may cause the difference in maturity times between MT and WT.

## Discussion

According to our results, the most active processes in fruit development, especially the early stage concentrated on the metabolic process and the regulation system, mainly including carbohydrate transport and metabolism, secondary metabolism-related processes, transcriptional modification, posttranslational modification and signal transduction (Fig. 3 and S1 Table). The findings of fruit ripening related processes are consistent with those of previous work [44]. Interestingly, the most clustered groups of DEGs also concentrated on the same processes (Fig. 5 and S3 Table). The findings of clustered groups of DEGs are in concordance with the research on grape development [45]. Most of the processes took place at the cellular level (Tables S1 and S2), suggesting that the biological processes at the cellular level are very important to plant development.

Among the 44,413 unigenes, 30.20% were differentially expressed in the two genotypes covering various biological pathways, indicating that the bud mutation caused a large-scale alteration in many biological processes. This result is in agreement with that of the research on red flesh mutant [22]. In addition, 75.8% of the DEGs were down-regulated in WT compared to MT (Fig. 4), indicating that the overall transcription level was lower in WT than in MT at the early ripening stage. The similar findings were reported on the transcriptome analyses of the fruit development of watermelon, date palm and the ‘Fengjie 72-1’ orange [8], [46], [47].

ABA participates in the regulation of fruit ripening in tomato [48], strawberry [26], grape [27] and bilberry [49]. This study reveals that *CsNCED1* plays the most important role in the ABA biosynthesis pathway during the fruit ripening process of Jincheng, because it has the highest expression level among the three ABA synthesis genes (Fig. 7). However, a previous research reported that in avocado, *NCED3* seems to be more important than *NCED1* in the ABA biosynthesis pathway during ripening process [50]. This study also reveals that the expression level of the genes involved in ABA biosynthesis and degradation increased with the ripening of fruit, suggesting that ABA mediates the feedback inhibition of its own biosynthesis. However, this result is in contrast with that of Jie Ren's research [51]. In their research, the expression level of ABA 8′-hydroxylase decreased during fruit ripening in the pulp of sweet cherry, indicating that the mechanism of ABA metabolism regulation is different in the different non-climacteric fruits. The expression patterns of the *PP2Cs* members in the fruits of the ‘Jincheng’ sweet orange were the same with those in tomato [48] and the ‘Navelate’ (*C. sinensis* L. Osbeck) orange [24]. The expression pattern present a rising tendency during fruit ripening. In addition, the majority of the expression patterns of *PYR/PYL* members and *SnRK2.2* increase in the ‘Jincheng’ sweet orange during fruit ripening but they decrease in tomato and the ‘Navelate’ orange. However, our results agree with those of the research on the fruit ripening of the ‘Fengjie 72-1’ orange [8]. Our results suggest that SnRK2.2 might promote PP2Cs by inhibiting PYR/PYL (Fig. 8), which would explain the reason why the overall expression level of *PYR/PYL*, *PP2Cs* and *SnRK2.2* increase during ripening.

It was reported that sucrose functioned in strawberry fruit ripening as a signal and via interaction with ABA [29], [30]. In this work, we analyzed two sucrose biosynthesis genes and three sucrose transporter genes (Fig. 9). The expression patterns of *CsSUC*s were similar to that of *NCED1*, suggesting the possible interaction of sucrose and ABA. A recent study on peach found that the expression level of most of the genes involved in ABA synthesis was correlated with the content of sucrose in fruit flesh, suggesting possible cross-talk between ABA and sucrose [52]. A study on the effect of ABA treatment on the fruit peel of the ‘Cara Cara’ Navel orange demonstrated that ABA treatment could significantly affect the glucose, fructose, sugar and total sugar content of the fruit, and different ABA concentration had different effects [53]. These findings suggest the crosstalk between ABA and sucrose.

The transcript levels of the JA metabolism and signal transduction pathways were analyzed throughout the citrus fruit ripening process for the first time. Three JA biosynthesis genes and three JA signal transduction genes were analyzed (Fig. 9). Their rising expression trend during citrus fruit ripening suggests their positive function in citrus fruit ripening. A study of strawberry fruit ripening demonstrated that JA could promote the ripening of fruits by getting involved in the processes of anthocyanin accumulation, cell wall modification and ethylene biosynthesis [31]. However, the study of peach demonstrated that early methyl jasmonate application to peach delayed the development of fruit and seed by altering the expression of multiple hormone-related genes [54], [55]. Another study on the effect of JA on tomato fruit ripening indicated that JA could accelerate fruit ripening by promoting the lycopene biosynthesis independently of ethylene [56]. In general, JA could affect the ripening process of fruit with or without interacting with other plant hormones. The effects of JA are different between climacteric fruits and non-climacteric fruits, and the effects of JA are different within climacteric fruits or non-climacteric fruits.

In total, the transcript level decreases during fruit ripening. ABA, sucrose, and JA could regulate sweet orange fruit ripening by interacting with each other (Fig. 8).

## Supporting Information

S1 Fig The secondary classification of the posttranslational modification (short for O, 357 DEGs). 1 Chaperones and folding catalysts; 2 Cysteine and methionine metabolism; 3 Electron transfer carriers; 4 Enzyme Families; 5 Glutathione metabolism; 6 Hydrolases; 7 Ligases; 8 Metabolism of Terpenoids and Polyketides; 9 Oxidative phosphorylation; 10 Proteasome; 11Protein folding and associated processing; 12 Replication and Repair; 13 Transferases; 14 Translation proteins; 15 Transport and Catabolism; 16 Two-component system; 17 Ubiquitin system; 18 Unclassified.
(TIF)

S2 Fig The secondary classification of translation (short for J, 322 DEGs). 1 Amino Acid Metabolism; 2 Aminoacyl-tRNA biosynthesis; 3 Cellular Processes and Signaling; 4 Metabolism of Terpenoids and Polyketides; 5 Nucleotide Metabolism; 6 Ribosome; 7 RNA transport; 8 Spliceosome; 9 Sulfur relay system; 10 Translation factors; 11 Unclassified.
(TIF)

S3 Fig The secondary classification of the carbohydrate transport and metabolism (short for G, 319 DEGs). 1 Amino sugar and nucleotide sugar metabolism; 2 Ascorbate and aldarate metabolism; 3 Carbon fixation in photosynthetic organisms; 4 Fructose and mannose metabolism; 5 Galactose metabolism; 6 Glucose metabolism; 7 Glycan Biosynthesis and Metabolism; 8 Glycolysis/Gluconeogenesis; 9 Golgi nucleoside diphosphatase; 10 Membrane Transport; 11 Metabolism of Cofactors and Vitamins; 12 Nitrogen metabolism; 13 Nucleotide Metabolism; 14 Pentose phosphate pathway; 15 Permeases of the drug/metabolite transporter (DMT) superfamily; 16 Permeases of the major facilitator superfamily; 17 Phosphoenolpyruvate synthase/pyruvate phosphate dikinase; 18 plasma membrane intrinsic protein; 19 Pyruvate metabolism; 20 Starch and sucrose metabolism; 21 Transferases; 22 Unclassified; 23 Xenobiotics Biodegradation and Metabolism.
(TIF)

S4 Fig The secondary classification of transcription (short for K, 267 DEGs). 1 tRNA processing pathway; 2 transport and catabolism; 3 transcription related proteins; 4 transcription factors; 5 spliceosome; 6 RNA polymerase; 7 replication and repair; 8 metabolism of cofactors and vitamins; 9 hydrolases; 10 heat shock transcription factor; 11 folding, sorting and degradation; 12 enzyme families.
(TIF)

S5 Fig The secondary classification of energy production and conversion (short for G, 297 DEGs). C Energy Metabolism; E Amino Acid Metabolism; F Nucleotide Metabolism; G Carbohydrate Metabolism; I Lipid Metabolism; P Transport and Catabolism; Q Biosynthesis of Other Secondary Metabolites; T Signaling; 1 Carbon fixation in photosynthetic organisms; 2 Methane metabolism; 3 Oxidative phosphorylation; 4 photosynthesis; 5 unclassified.
(TIF)

S6 Fig The secondary classification of the signal transduction mechenisms (short for T, 171 DEGs). 1 Calcium signaling pathway; 2 Cell Growth and Death; 3 Circadian rhythm – plant; 4 Enzyme Families; 5 mTOR signaling pathway; 6 Genetic Information Processing; 7 Glycerolipid metabolism; 8 Indole alkaloid biosynthesis; 9 Lipid Metabolism; 10 Neurotrophin signaling pathway; 11 Phosphatidylinositol signaling system; 12 Plant hormone signal transduction; 13 Plant-pathogen interaction; 14 Transferases; 15 Translation; 16 Unclassified; 17 Wnt signaling pathway.
(TIF)

S7 Fig The secondary classification of secondary metabolites biosynthesis, transport and catabolism (short for Q, 137 DEGs). 1 Ascorbate and aldarate metabolism; 2 Benzoxazinoid biosynthesis; 3 Enzyme Families; 4 Flavonoid biosynthesis; 5 Genetic Information Processing; 6 Isoquinoline alkaloid biosynthesis; 7 Lipid Metabolism; 8 Metabolism of Cofactors and Vitamins; 9 Metabolism of Terpenoids and Polyketides; 10 Nicotinate and nicotinamide metabolism; 11 Phenylpropanoid biosynthesis; 12 Porphyrin and chlorophyll metabolism; 13 Transport and Catabolism; 14 Xenobiotics Biodegradation and Metabolism.
(TIF)

S1 Table All-unigene classification: The clusters of orthologous groups (COG), Gene Ontology (GO) classification, and Kyoto encyclopedia of genes and genomes (KEGG) analysis of all the unigenes are shown.
(XLS)

S2 Table Differently expressed unigenes (DEGs) list: The length and distribution in the wild type and the mutant, and difference rations are shown for all of the DEGs.
(XLS)

S3 Table Differently expressed unigenes (DEGs) classification: The Gene Ontology (GO) classification and the Kyoto encyclopedia of genes and genomes (KEGG) analysis of all the DEGs.
(XLS)

S4 Table Annotation of DEGs with at least three database hits.
(XLS)

S5 Table Blast searching against the *Citrus sinensis* CDS Database published by Qiang Xu et al.: The sequences of all of the unigenes were blasted against the published *Citrus sinensis* CDS sequences with an e-value of le-5. The data show all of the hits with an identity of greater than 95%.
(XLS)

S6 Table Blast searching against the *Citrus sinensis* CDS Database published by Fred G. Gmitter et al.: The sequences of all of the unigenes were blasted against the published *Citrus sinensis* CDS sequences with an e-value of le-5. The data show all of the hits with an identity of greater than 95%.
(XLS)
