# Supplementary figures and images for: Comparative Transcriptome Analyses between a Spontaneous Late-Ripening Sweet Orange Mutant and Its Wild Type Suggest the Functions of ABA, Sucrose and JA during Citrus Fruit Ripening
Source: PLoS One. 2014 Dec 31;9(12):e116056. doi: 10.1371/journal.pone.0116056 (PMC4281182; doi:10.1371/journal.pone.0116056)

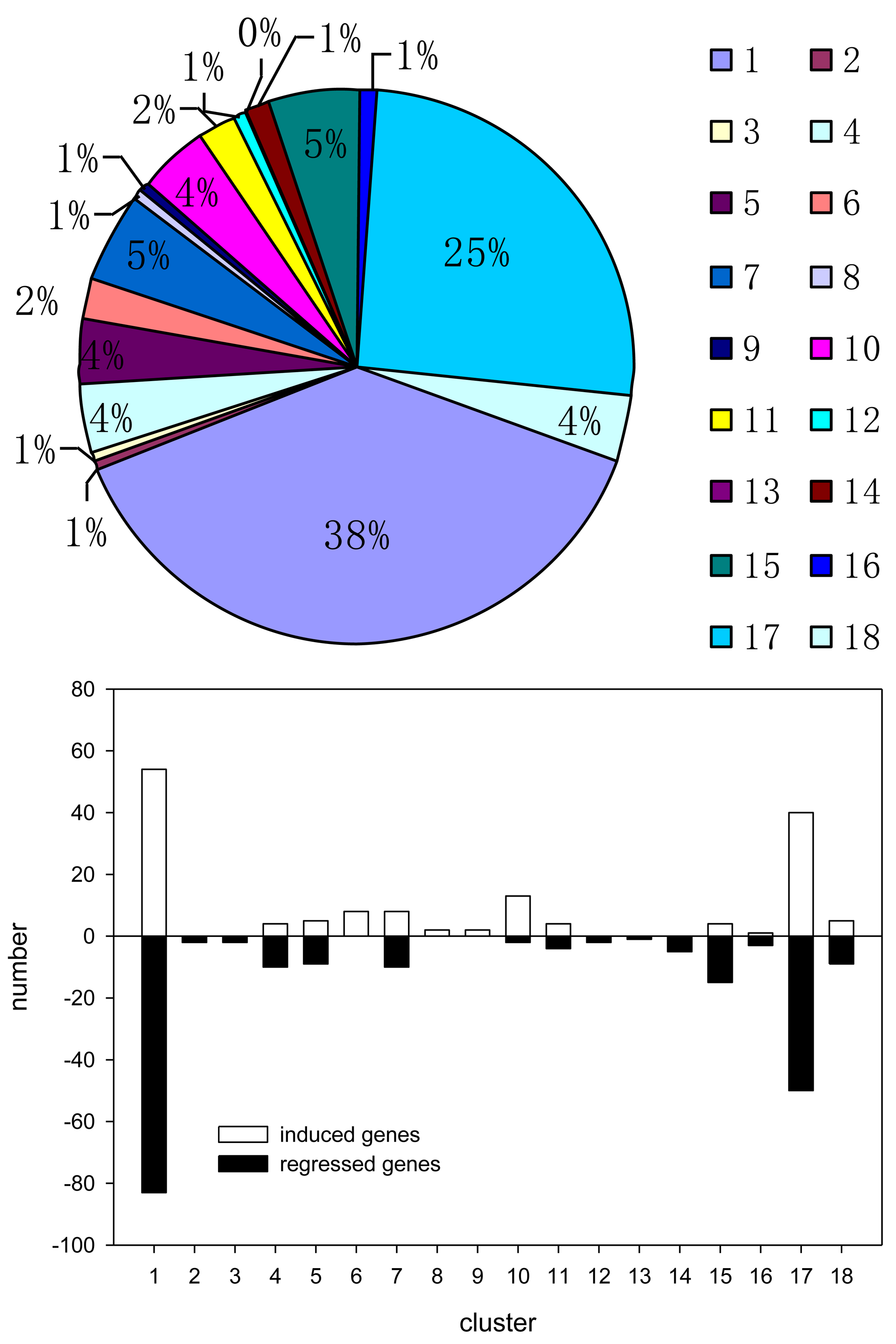

Supplement: S1 Fig — The secondary classification of the posttranslational modification (short for O, 357 DEGs). 1 Chaperones and folding catalysts; 2 Cysteine and methionine metabolism; 3 Electron transfer carriers; 4 Enzyme Families; 5 Glutathione metabolism; 6 Hydrolases; 7 Ligases; 8 Metabolism of Terpenoids and Polyketides; 9 Oxidative phosphorylation; 10 Proteasome; 11Protein folding and associated processing; 12 Replication and Repair; 13 Transferases; 14 Translation proteins; 15 Transport and Catabolism; 16 Two-component system; 17 Ubiquitin system; 18 Unclassified. (TIF) [file pone.0116056.s001.tif]

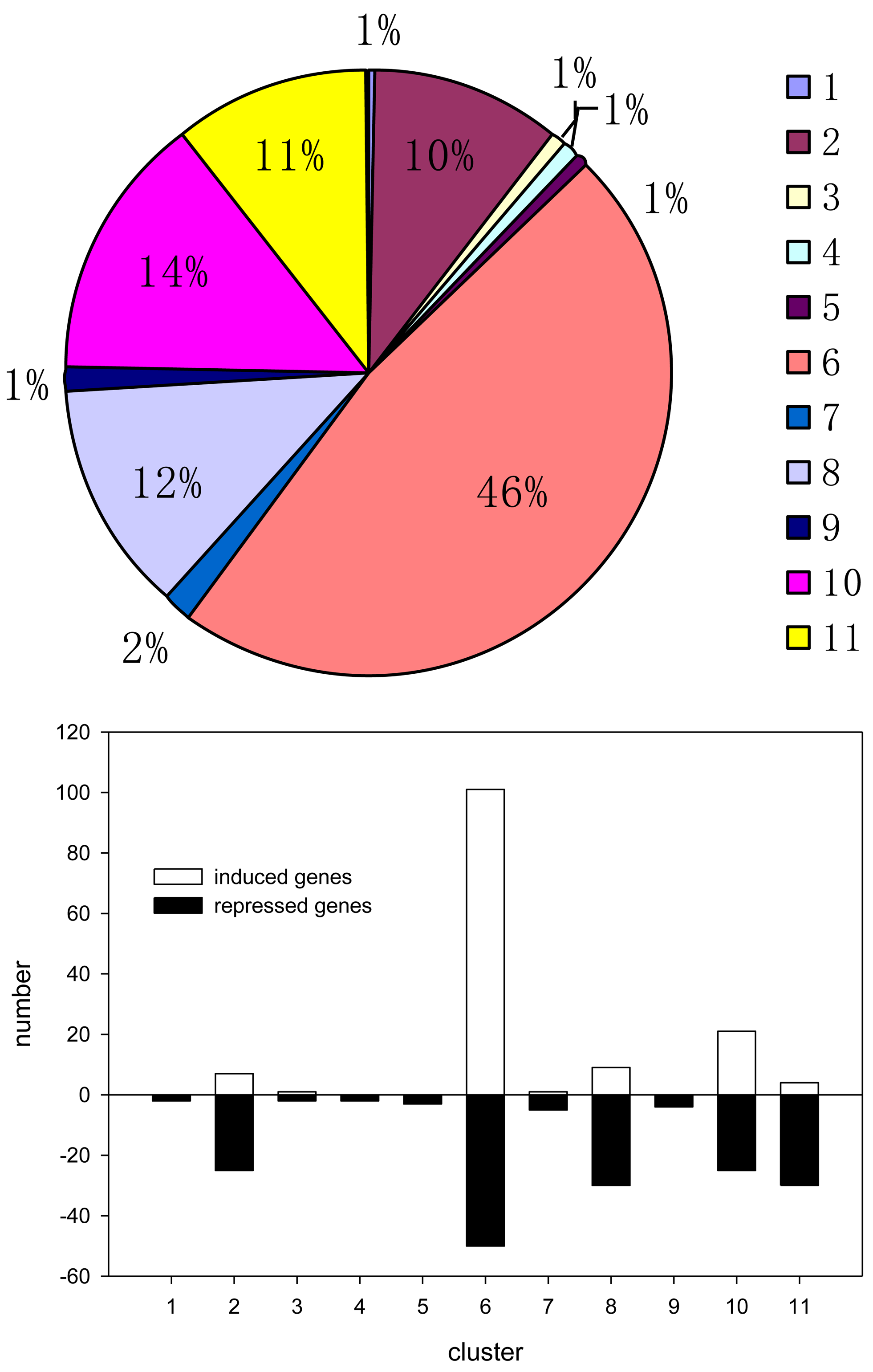

Supplement: S2 Fig — The secondary classification of translation (short for J, 322 DEGs). 1 Amino Acid Metabolism; 2 Aminoacyl-tRNA biosynthesis; 3 Cellular Processes and Signaling; 4 Metabolism of Terpenoids and Polyketides; 5 Nucleotide Metabolism; 6 Ribosome; 7 RNA transport; 8 Spliceosome; 9 Sulfur relay system; 10 Translation factors; 11 Unclassified. (TIF) [file pone.0116056.s002.tif]

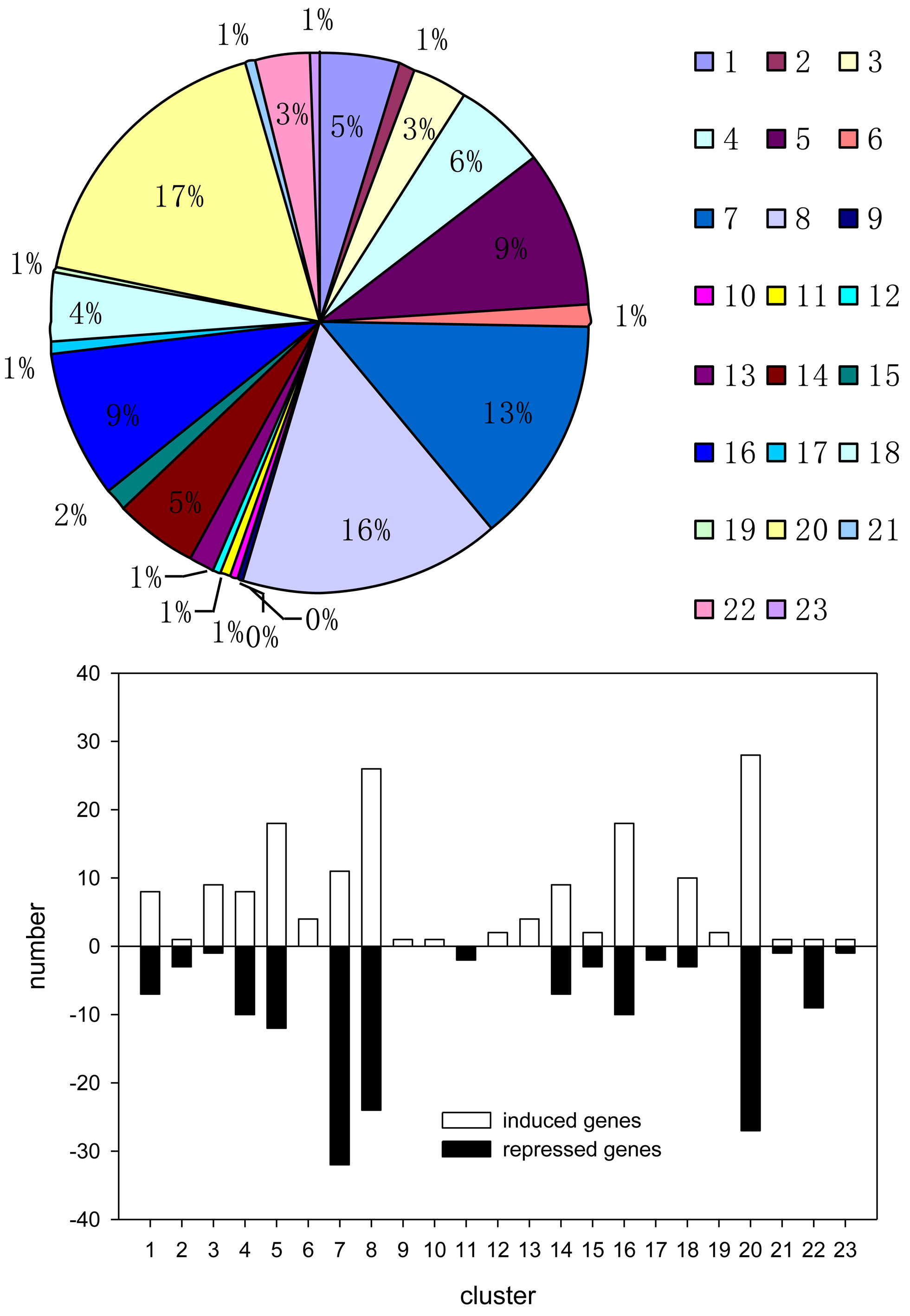

Supplement: S3 Fig — The secondary classification of the carbohydrate transport and metabolism (short for G, 319 DEGs). 1 Amino sugar and nucleotide sugar metabolism; 2 Ascorbate and aldarate metabolism; 3 Carbon fixation in photosynthetic organisms; 4 Fructose and mannose metabolism; 5 Galactose metabolism; 6 Glucose metabolism; 7 Glycan Biosynthesis and Metabolism; 8 Glycolysis/Gluconeogenesis; 9 Golgi nucleoside diphosphatase; 10 Membrane Transport; 11 Metabolism of Cofactors and Vitamins; 12 Nitrogen metabolism; 13 Nucleotide Metabolism; 14 Pentose phosphate pathway; 15 Permeases of the drug/metabolite transporter (DMT) superfamily; 16 Permeases of the major facilitator superfamily; 17 Phosphoenolpyruvate synthase/pyruvate phosphate dikinase; 18 plasma membrane intrinsic protein; 19 Pyruvate metabolism; 20 Starch and sucrose metabolism; 21 Transferases; 22 Unclassified; 23 Xenobiotics Biodegradation and Metabolism. (TIF) [file pone.0116056.s003.tif]

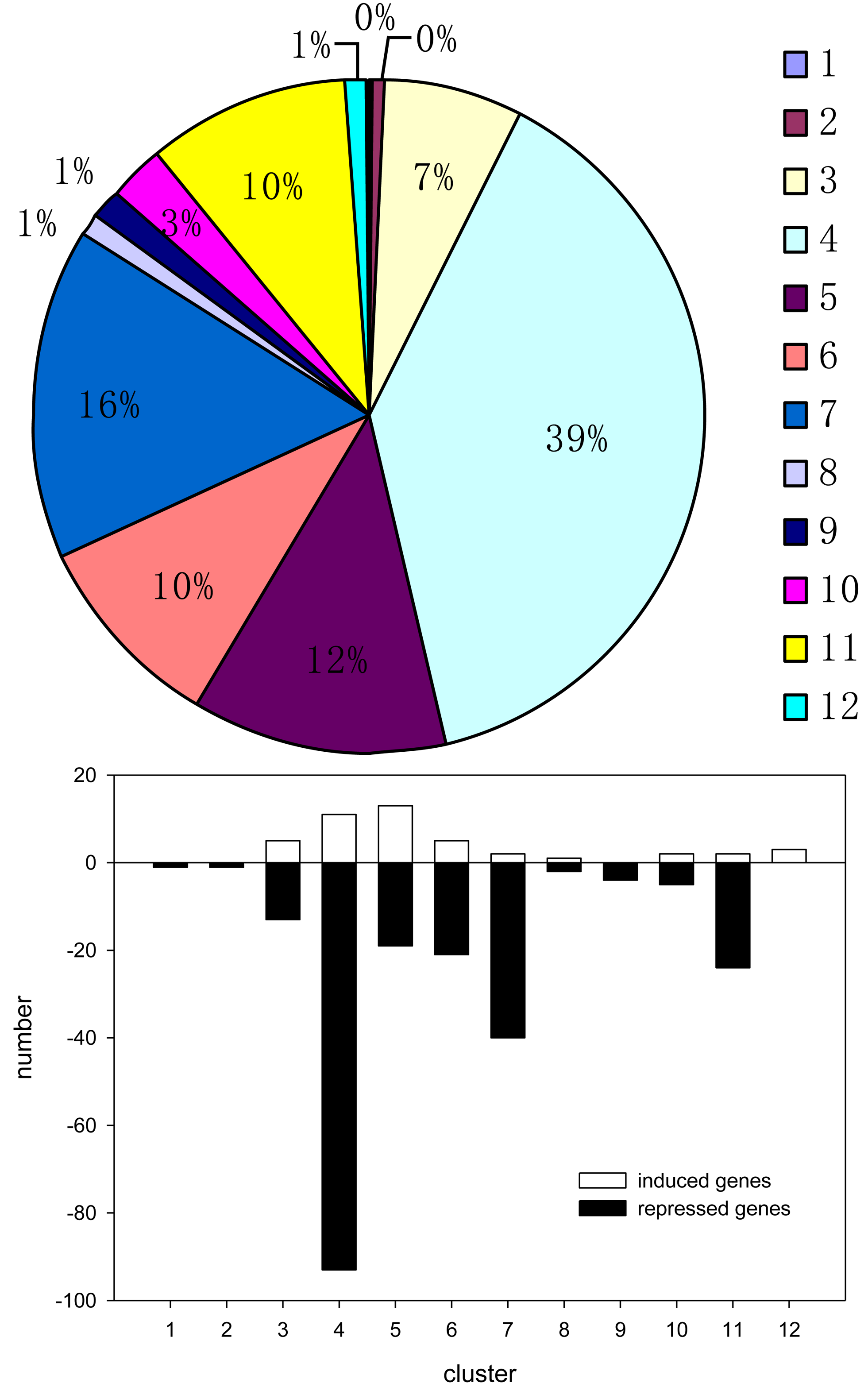

Supplement: S4 Fig — The secondary classification of transcription (short for K, 267 DEGs). 1 tRNA processing pathway; 2 transport and catabolism; 3 transcription related proteins; 4 transcription factors; 5 spliceosome; 6 RNA polymerase; 7 replication and repair; 8 metabolism of cofactors and vitamins; 9 hydrolases; 10 heat shock transcription factor; 11 folding, sorting and degradation; 12 enzyme families. (TIF) [file pone.0116056.s004.tif]

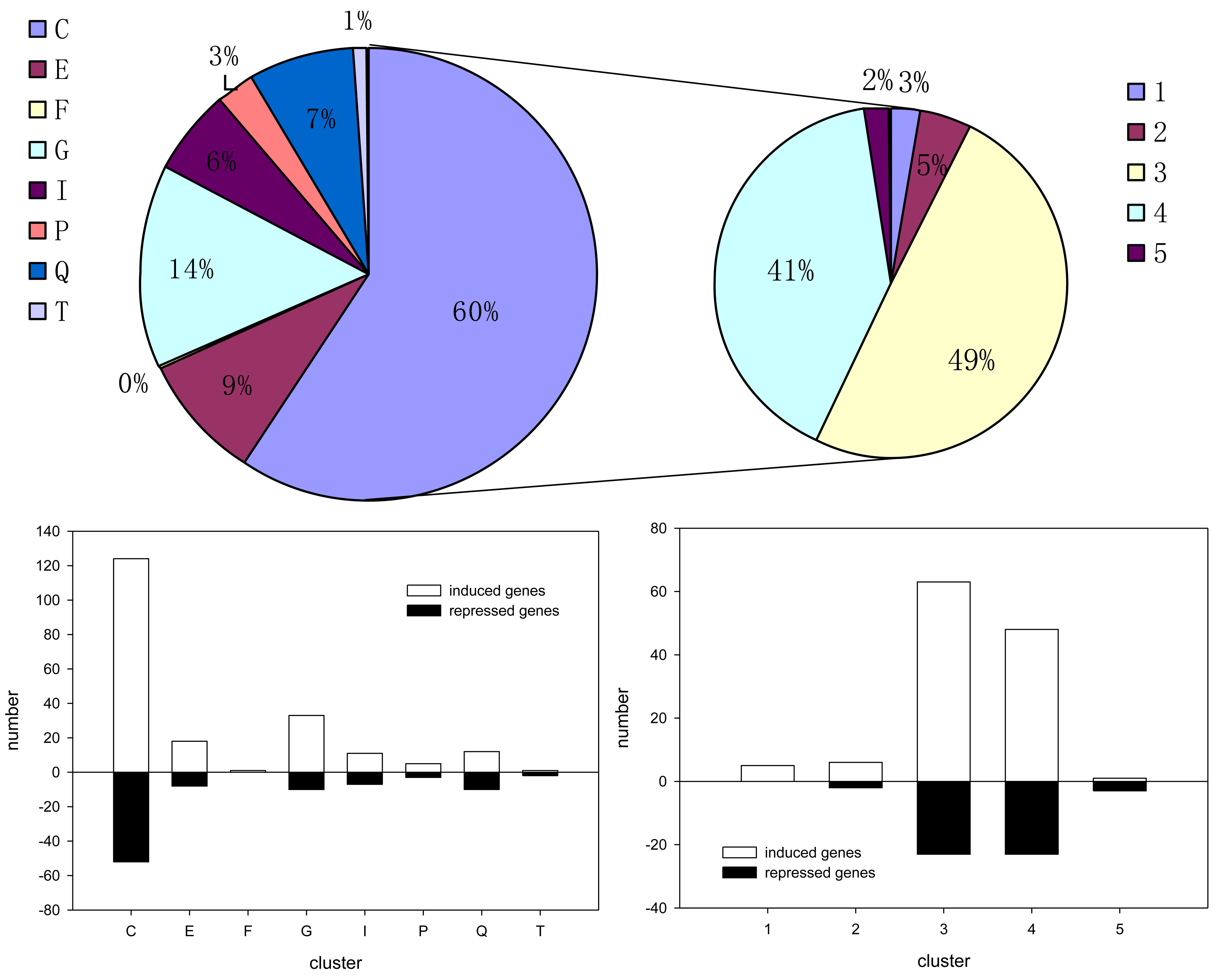

Supplement: S5 Fig — The secondary classification of energy production and conversion (short for G, 297 DEGs). C Energy Metabolism; E Amino Acid Metabolism; F Nucleotide Metabolism; G Carbohydrate Metabolism; I Lipid Metabolism; P Transport and Catabolism; Q Biosynthesis of Other Secondary Metabolites; T Signaling; 1 Carbon fixation in photosynthetic organisms; 2 Methane metabolism; 3 Oxidative phosphorylation; 4 photosynthesis; 5 unclassified. (TIF) [file pone.0116056.s005.tif]

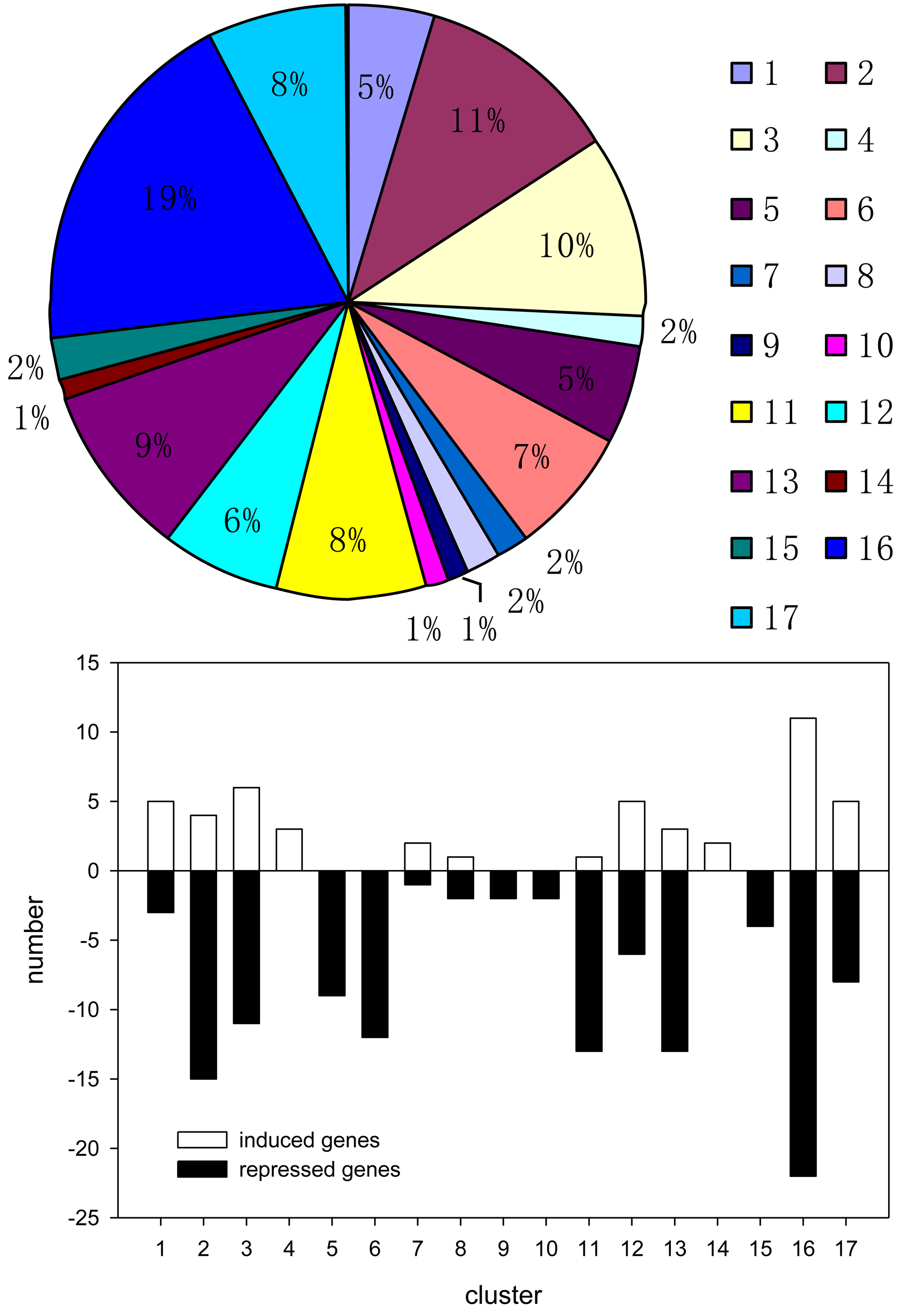

Supplement: S6 Fig — The secondary classification of the signal transduction mechenisms (short for T, 171 DEGs). 1 Calcium signaling pathway; 2 Cell Growth and Death; 3 Circadian rhythm – plant; 4 Enzyme Families; 5 mTOR signaling pathway; 6 Genetic Information Processing; 7 Glycerolipid metabolism; 8 Indole alkaloid biosynthesis; 9 Lipid Metabolism; 10 Neurotrophin signaling pathway; 11 Phosphatidylinositol signaling system; 12 Plant hormone signal transduction; 13 Plant-pathogen interaction; 14 Transferases; 15 Translation; 16 Unclassified; 17 Wnt signaling pathway. (TIF) [file pone.0116056.s006.tif]

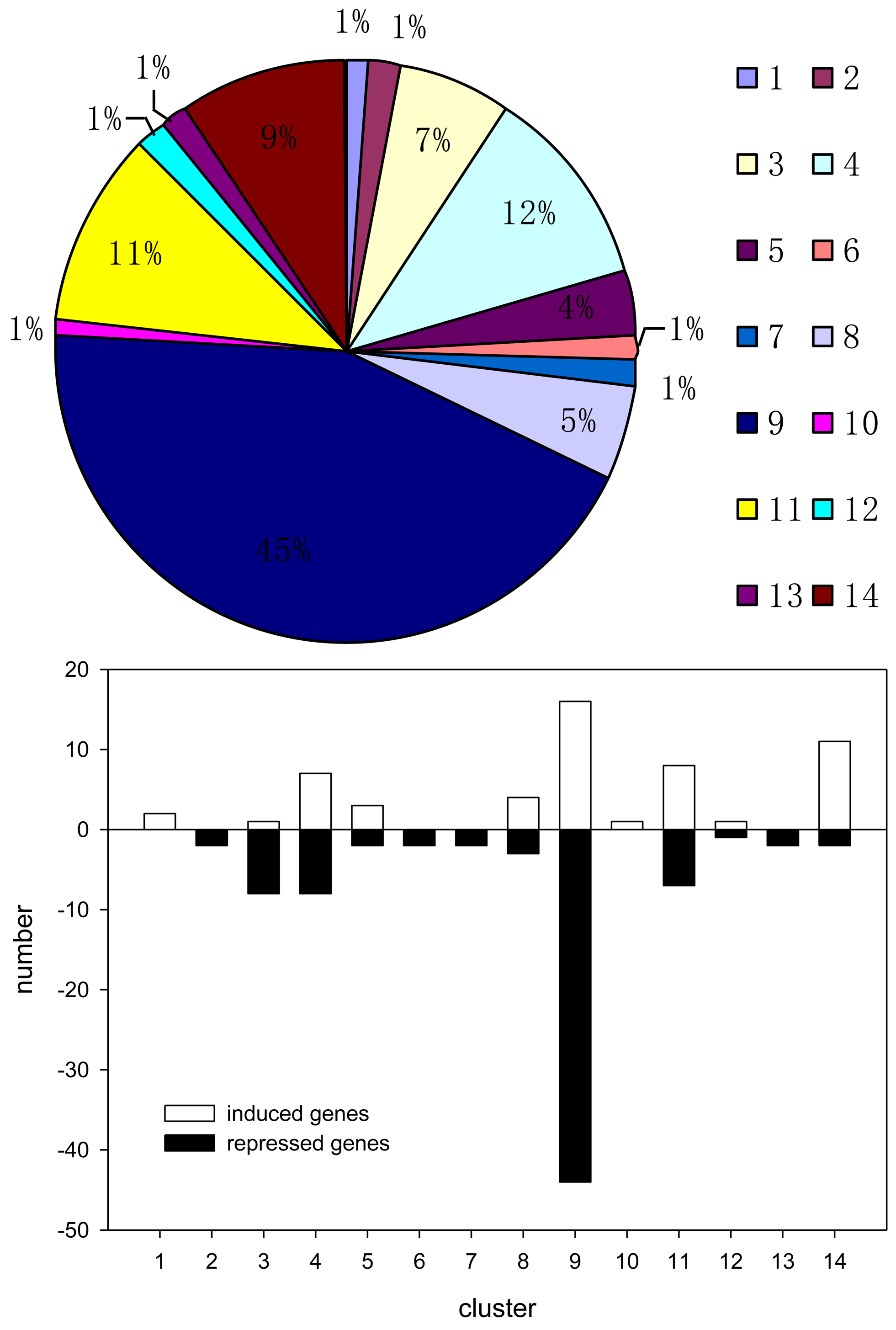

Supplement: S7 Fig — The secondary classification of secondary metabolites biosynthesis, transport and catabolism (short for Q, 137 DEGs). 1 Ascorbate and aldarate metabolism; 2 Benzoxazinoid biosynthesis; 3 Enzyme Families; 4 Flavonoid biosynthesis; 5 Genetic Information Processing; 6 Isoquinoline alkaloid biosynthesis; 7 Lipid Metabolism; 8 Metabolism of Cofactors and Vitamins; 9 Metabolism of Terpenoids and Polyketides; 10 Nicotinate and nicotinamide metabolism; 11 Phenylpropanoid biosynthesis; 12 Porphyrin and chlorophyll metabolism; 13 Transport and Catabolism; 14 Xenobiotics Biodegradation and Metabolism. (TIF) [file pone.0116056.s007.tif]
